# Dynamical analysis of an inverted pendulum with positive position feedback controller approximate uniform solution

**DOI:** 10.1038/s41598-023-34918-x

**Published:** 2023-05-31

**Authors:** Galal M. Moatimid, A. T. El-Sayed, Hala F. Salman

**Affiliations:** 1 Department of Mathematics, Faculty of Education, Ain Shams University, Cairo, Egypt; 2 Department of Basic Science, Modern Academy for Engineering and Technology, Cairo, Egypt; 3 Department of Basic Sciences, Faculty of Computers and Artificial Intelligence, Cairo University, Giza, Egypt

**Keywords:** Engineering, Mathematics and computing, Physics

## Abstract

The inverted pendulum is controlled in this article by using the nonlinear control theory. From classical analytical mechanics, its substructure equation of motion is derived. Because of the inclusion of the restoring forces, the Taylor expansion is employed to facilitate the analysis. An estimated satisfactory periodic solution is obtained with the aid of the modified Homotopy perturbation method. A numerical technique based on the fourth-order Runge–Kutta method is employed to justify the previous solution. On the other hand, a positive position feedback control is developed to dampen the vibrations of an IP system subjected to multi-excitation forces. The multiple time scale perturbation technique of the second order is introduced as a mathematical method to solve a two-degree-of-freedom system that simulates the IP with the PPF at primary and 1:1 internal resonance. The stability of these solutions is checked with the aid of the Routh–Hurwitz criterion. A set of graphs, based on the frequency response equations resulting from the MSPT method, is incorporated. Additionally, a numerical simulation is set up with RK-4 to confirm the overall controlled performance of the studied model. The quality of the solution is confirmed by the match between the approximate solution and the numerical simulation. Numerous other nonlinear systems can be controlled using the provided control method. Illustrations are offered that pertain to implications in design and pedagogy. The linearized stability of IP near the fixed points as well as the phase portraits is depicted for the autonomous and non-autonomous cases. Because of the static stability of the IP, it is found that its instability can be suppressed by the increase of both the generalized force as well as the torsional constant stiffness of the spring. Additionally, the presence of the magnetic field enhances the stability of IP.

## Introduction

The IP has received a lot of attention in recent years because of two essential components: high ordering and significant relationship, both of which are significant concerns in the control area. Moreover, the mechanism is unstable, nonlinear, and multivariable. Many processes in the military, aircraft, robotics, and general industries can benefit from using IP control techniques, including those that deal with robot walking balance challenges, rocket launch important considerations, and satellite flying control problems. Thus, it is extremely important to use this approach to investigate the IP scheme^1–3^. Furthermore, the mechanism for producing force based on the electromagnetic impact to preserve the IP in balancing is produced in the IP system by applying the force to the upper end of the IP and the electromagnetic influence on the IP. The IP is used in numerous engineering applications, including single-wheeled mobile robots, personal transporters, and walking robots^4^. Balanced management of an IP is a daunting challenge because the dynamics of IP are fundamentally open-loop unstable, nonlinear, underactuated, and non-minimum phase. It is frequently used as a reference point for developing and approving various control systems. The IP has been widely utilized in controlling education for many years due to its significance in control engineering.

Analytical calculations and actual observations were used to examine the nonlinear vibrational behavior of a linearly regulated rotary IP^5^. The fundamental coupled nonlinear equations of the structure, which were developed employing Lagrange's variational principle, were solved by developing the MSPT. Among the traditional systems where parameterized instability develops is the vertically propelled pendulum. Whenever the bob is a magnetic dipole, an extra electromagnetic relationship created by eddy currents in a neighboring thick conducting plate was investigated^6^. Studies addressing the scientific framework, recommending a hybrid control technique, and creating a workable model of an IP were examined^7^. Results from simulations and actual experiments were achieved, confirming the IP model performance and adaptability when combined with a suitable hybrid control method in a balanced situation. The IP was a straightforward and useful laboratory simulation of a mechanical system that was unstable. The explanation of the historical development of IP and the contrast between a number of its earlier procedures, which were carried out between 1960 and 1970 according to the earlier bibliography, were examined^8^. In order to comprehend how design decisions, affect balancing effectiveness, five areas of development for wheeling IP systems were examined^9^. Elasticity needs to be incorporated into mathematical models for the system of dynamics analysis. Elasticity was taken into consideration when a new type of IP, the elastic IP, was developed^10^. For a reaction wheel IP subject to biased angle measurements, a linear analysis was developed^11^. In this study, the stability issue was considered when there was an ongoing, unidentified bias in measurements of the pendulum angle. It has significant practical implications because it permits both a closer approximation of the control of robots and a less exact arrangement of sensors. A generalized mathematical model of IP was used to study how the pendulum factors and follower force affect the development of equilibrium situations. The parameter extension approach was used to plot equilibrium curves^12^.

Numerous engineering and physical phenomena are described via nonlinear differential equations. Most of those concerns, with the exception of a few numbers, lack obtaining an exact analytical solution. Really, this requires approximate methods to determine such equations **b**ecause the HPM^13^ has better quality calculation than the earlier techniques. Additionally, it is a straightforward, strong, efficient, attractive, powerful, and promising approach. Therefore, scientists and engineers have given much attention to this methodology in application in nonlinear problems. The Laplace transforms dispersive heat radiation, integral heat conduction, nonlinear oscillators, nonlinear Schrodinger equations, nonlinear chemistry, and many other nonlinear issues have already been addressed by using the HPM^14^. In most situations, it results in a very quick convergence of the series solution, typically with just a few iterations leading to highly precise solutions. Consequently, HPM is efficient in resolving many types of nonlinear equations. A minor equational parameter has no bearing on the technique. The homotopy was built using the homotopy procedure in topology with an embedded parameter $\rho \in \left[ {0,\,1} \right]$ that was regarded as a "small parameter". It has been used to approximately solve a wide range of nonlinear problems efficiently, quickly, and appropriately. These approximations quickly reach the correct answers as found earlier in^15,16^. A hybrid Rayleigh–Van der Pol–Duffing oscillator with an exciting exterior force and nonlinear terms was studied^17^. To reach an approximate solution, the method of Poincaré–Lindstedt was adopted during their approach.

In order to demonstrate that the vibration induced by the external force was suppressed, a time history comparison was made for both the uncontrolled and the controlled models. Three different types of controllers were added to study the best of them in damping the vibration occurring in the harmonic force Duffing oscillator system^18^. They used the MSPT to find the approximate solution for this system after connecting it to the nonlinear integrated positive position feedback (NIPPF) controller. The PPF controller dampens the vibration that occurred in a nonlinear exciting beam. To clarify the best conditions to reduce this vibration, they examined the effect of different parameters in the resonance case^19^. The influence of the PPF technique was utilized to analyze a solar panel prototype^20^. On the other hand, EL-Ganaini et al.^21^ used a PPF control to decrease the nonlinear vibration of a dynamical framework within a 1:1 internal resonance. The approximate solution was achieved by applying the MSPT. Moreover, EL-Sayed^22^ investigated a pair of delay PPF control that can minimize the vibration of double Van der Pol oscillators with external forces. The impacts of both the delayed feedback signal and the control gains were studied to demonstrate the low vibration amplitudes.

In light of the above-mentioned aspects, the current paper focuses on examining the problem of the motion of an IP. Furthermore, the main aim of this study is to suppress the harmful vibration in the IP system by using a PPF controller which has also been used in the previous references. The rest of the manuscript is structured as follows: The methodology of the problem is described in section "Methodology of the problem". In view of the HPM and the idea of the extended nonlinear frequency, an approximate uniform solution to the problem is described in section "Expanded frequency analysis". Section "Linearized stability of the autonomous equation" is dedicated to illustrating the linearized problem. The IP system with the controller is applied in section "IP system with PPF controller". The frequency response equations (FREs) and stability studies are shown in section "Linearized stability of the non-autonomous equation". Section "Results and discussions" introduces the results and discussions for the controller design. Finally, section "Conclusions" summarizes the key results.

## Methodology of the problem

**Figure 1:**
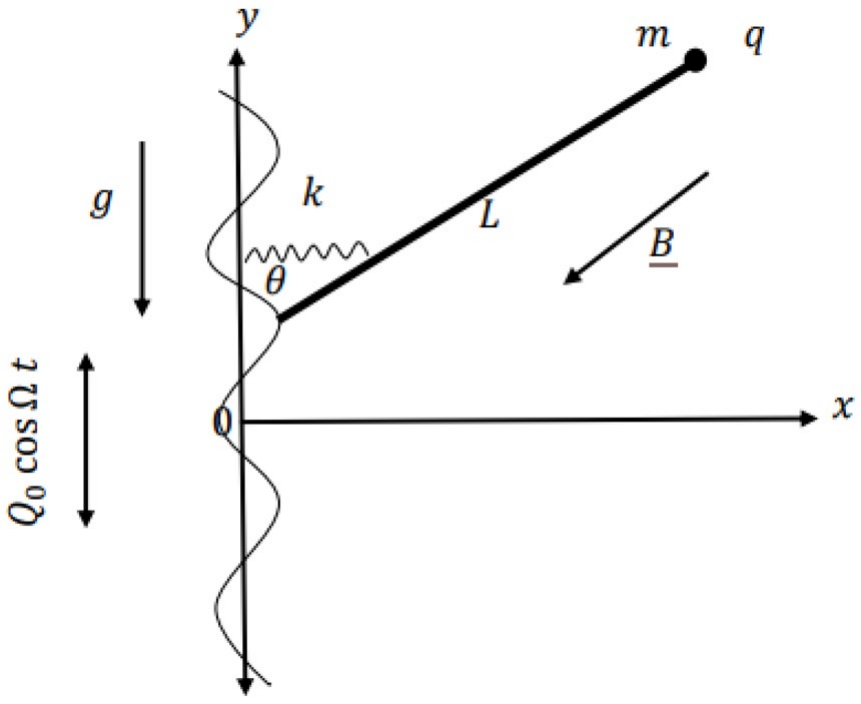
Sketches the IP with a vertical periodic moving base.

A magnetic IP is regarded as a mechanical system, where its bob has a point mass $m$ and an electrical charge $e$. It is connected to the length $L$ of the weightless stiff rod. The other end of the rod swings vertically with a periodic displacement $Q_{0} \cos \Omega t$ where $Q_{0}$ and $\Omega$ are the movement amplitude and frequency, respectively. The IP revolves around a fixed point $0$. It rotates under the influence of a constant gravity force $\underline{g}$ and a steady negative oriented magnetic field $\underline{B}$ that acts in the negative *z*-direction. Because of the strong instability of the configuration of the IP, a light torsional spring of constant stiffness $k$ is attached between its base and the *y*-axis. The sketch of the theoretical prototype of the IP may be exemplified in Fig. 1.

**Figure 2:**
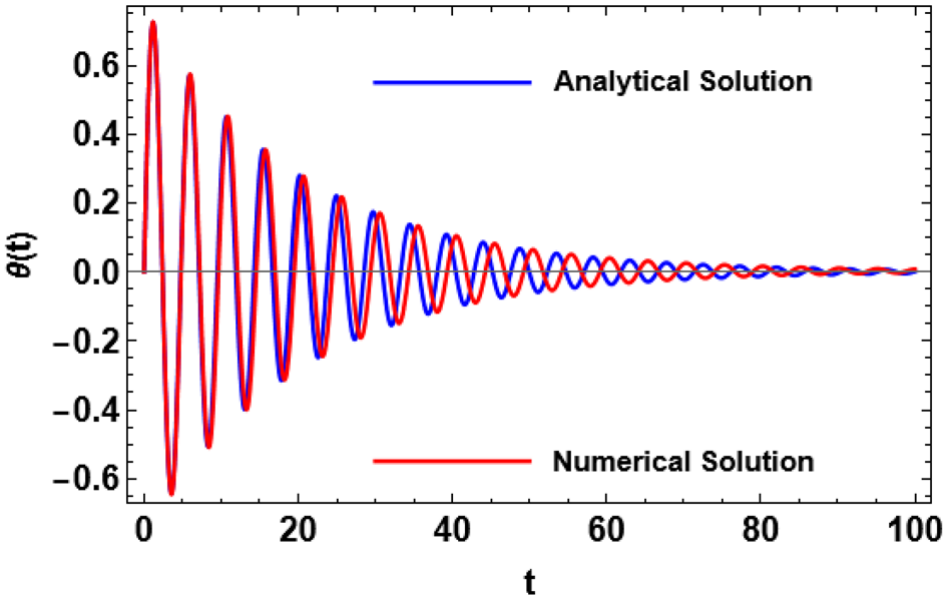
Perturbed/numerical solutions of Eq. (14).

As shown in this figure, the position vector of the bob of the IP is given as:

1 $$\underline{r} = L\,\sin \theta \,\underline{i} + (L\cos \theta + Q_{0} \cos \Omega t\,)\underline{j} ,$$

where $\theta$ is measured between the IP and the *y*-axis.

It follows that velocity may be given by:

2 $$\underline{v} = L\dot{\theta }\,\cos \theta \,\underline{i} - (L\dot{\theta }\sin \theta \, + \Omega \,Q_{0} \sin \Omega t)\underline{j} ,$$

Subsequently, the kinetic energy becomes:

3 $$T = \frac{m}{2}\left[ {L^{2} \dot{\theta }^{2} + 2L\Omega Q_{0} \dot{\theta }\sin \theta \sin \Omega t + \Omega^{2} Q_{0}^{2} \sin^{2} \Omega t} \right].$$

The potential energy probably originated from a number of actions, which can be grouped into the following categories:

- The energy in the relation to a gravitational field, or gravitational potential energy has a definition of $mgL\cos \theta$.
- The potential energy stored as a result of flexible body deformation, such as a spring expanding, is known as elastic energy. The effort put forth in stretching the spring could serve as its metaphor. It is based on the constricted length and the constant stiffness of the spring $k$. It can be written as $k\theta^{2} /2$.
- The potential function of the issue, therefore, must include the magnetic term $q\,\underline{P} \,.\,\underline{v}$, where $\underline{P}$ is the magnetic vector potential**;** for example, see Eyal and Goldstein^23^, due to the charged IP traveling in a homogeneous magnetic field $\underline{B} = B\,\underline{k}$. Remember that it determines the connection between the magnetic field $\underline{B}$ and the magnetic potential $\underline{P}$ and is given by $\underline{B} = \nabla \wedge \underline{P}$. The practical significance of a homogenous magnetic field is widely established. One may demonstrate that one of the vector potential alternatives is provided by $\underline{P} = \tfrac{1}{2}\left( {\underline{B} \wedge \underline{r} } \right)$. The sentence that follows can be expressed as follows^24^:
  4 $$\underline{P} = \tfrac{1}{2}B\left( {( - L\cos \theta - Q_{0} \cos \Omega t)\,\underline{i} + L\sin \theta \,\underline{j} } \right).$$

The potential energy caused by the uniform magnetic field can be expressed by merging Eqs. (2) and (4) as follows:

5 $$\underline{P} .\,\underline{E}_{mag} = - \tfrac{1}{2}q\,L\,B\left[ {\left. {L\dot{\theta } + Q_{0} \,\dot{\theta }\,\cos \theta \cos \Omega t + \Omega \,Q_{0} \sin \theta \sin \Omega t} \right]} \right.,$$

Accordingly, the formulation for the total expected energy is as follows:

6 $$V = m\,g\,L\cos \theta + \tfrac{1}{2}k\,\theta^{2} - \tfrac{1}{2}q\,L\,B\left[ {L\dot{\theta } + Q_{0} \,\dot{\theta }\cos \theta \cos \Omega t + \Omega \,Q_{0} \sin \theta \sin \Omega t} \right].$$

Incorporating Eqs. (3) and (6), it is evident that the magnetic IP under consideration has the following Lagrangian function:

7 $$\begin{aligned} \Re & = T - V \\ & = \tfrac{1}{2}m\,\left[ {L^{2} \dot{\theta }^{2} + 2L\Omega Q_{0} \dot{\theta }\sin \theta \sin \Omega t + \Omega^{2} Q_{0}^{2} \sin^{2} \Omega t} \right] \\ & \,\,\,\,\,\, - \left\{ {m\,g\,L\cos \theta + \tfrac{1}{2}k\,\theta^{2} - \tfrac{1}{2}q\,LB\left( {L\dot{\theta } + Q_{0} \,\dot{\theta }\cos \theta \cos \Omega t + \Omega \,Q_{0} \sin \theta \sin \Omega t} \right)} \right\}. \\ \end{aligned}$$

However, the force under consideration becomes a non-conservative force when the air-dampening force is considered. This force can be expressed in the following way:

8 $$\underline{G}_{D} = - \delta \,\underline{v} .$$

Therefore, one gets

9 $$\underline{G}_{D} = \delta \left( {( - L\dot{\theta }\,\cos \theta )\,\underline{i} + (L\dot{\theta }\sin \theta + \Omega \,Q_{0} \sin \Omega t\,\underline{)j} } \right).$$

The structure has only one degree of freedom, and the generalized coordinate is expressed by an angle of measure $\theta$. In addition to this explanation, the generalized force is described as:

10 $$Q = \underline{G}_{D} \,.\,\frac{{\partial \underline{r} }}{\partial \theta } = - \delta \,L^{2} \dot{\theta } - \delta \,\Omega \,LQ_{0} \sin \theta \sin \Omega t.$$

From the analytical mechanics perspective, Lagrange’s equation with the non-conservative system may be written as^25^:

11 $$\frac{d}{dt}\left( {\frac{\partial \Re }{{\partial \dot{\theta }}}} \right) - \frac{\partial \Re }{{\partial \theta }} = Q.$$

When Eqs. (7) and (10) are inserted into Eq. (11), the controlling dynamic model of the magnetic IP under consideration can be described as follows:

12 $$m\,L^{2} \ddot{\theta } + \delta \,L^{2} \dot{\theta } + k\theta + L\,(m\,\Omega^{2} Q_{0} \cos \Omega t - mg + \delta \,\Omega \,Q_{0} \sin \Omega t)\,\sin \theta - L\,q\,B\,Q_{0} \,\Omega \,\sin \Omega t\,\cos \theta = 0.$$

A second-order differential equation with a harmonic restoring force and multiple parameterized forces is represented by Eq. (12).

Now let us go back to the initial controlling equation found in Eq. (12). The stability requirements of the magnetic IP under consideration will be analyzed using a unique technique in the following sections. A non-dimensional approach will be required before dealing with the quantitative equations of the governing equation of the IP as stated in Eq. (12), for more convenience. For this purpose, the non-dimensional technique is performed in a wide range of ways which largely depend on the characteristics of length, duration, and mass. In the problem under consideration, the characteristics are taken as follows $Q_{0} ,\,\,\sqrt {Q_{0} /g}$ and $m$. Therefore, Eq. (12) may be transformed into the reduced communication form in addition to these features:

13 $$\,L^{2} \ddot{\theta } + \delta \,L^{2} \dot{\theta } + k\theta + L\,(\,\Omega^{2} \cos \Omega t - 1 + \delta \,\Omega \sin \Omega t)\,\sin \theta - L\,H\,\Omega \,\,\sin \Omega t\,\cos \theta = 0.$$

where the non-dimensional term of the magnetic contributions $H$, which results from the multiplication $qB$, is referred to as the parameterization of the magnetic field.

## Expanded frequency analysis

The primary aim of this Section is to achieve a circumscribed analytical approximate solution for the second-order differential as given in Eq. (13). For this purpose, we will use an approximation of the circle functions as: $\sin \,\theta \approx \theta - \frac{1}{6}\theta^{3} + ...$ and $\cos \,\theta \approx 1 - \frac{1}{2}\theta^{2} + ...$. Therefore, Eq. (13) then becomes:

14 $$\ddot{\theta } + \delta \,\dot{\theta } + \omega^{2} \theta - \frac{\Omega \,H}{L}\,\sin \Omega t\,\,\left( {1 - \frac{{\theta^{2} }}{2}} \right) - \left( { - 1 + \Omega^{2} \cos \Omega t + \delta \,\Omega \,\sin \Omega t} \right)\,\frac{{\theta^{3} }}{6\,L} + \left( {\Omega^{2} \cos \Omega t + \delta \,\Omega \,\sin \Omega t} \right)\,\frac{\theta }{\,L} = 0,$$

where the natural frequency of the current prototype becomes $\omega^{2} = \frac{1}{{L^{2} }}\left( {k - L} \right)$.

The following performance is primarily dependent on a few HPM and Laplace transforms ($L_{T}$)**.** Equation (14) may be then interpreted as:

15 $$I(\theta ) = \ddot{\theta } + \omega^{2} \,\theta ,$$

and

16 $$N(\theta ) = \delta \,\dot{\theta } - \frac{\Omega \,H}{L}\,\sin \Omega t\,\,\left( {1 - \frac{{\theta^{2} }}{2}} \right) - \left( { - 1 + \Omega^{2} \cos \Omega t + \delta \,\Omega \,\sin \Omega t} \right)\,\frac{{\theta^{3} }}{6\,L} + \left( {\Omega^{2} \cos \Omega t + \delta \,\Omega \,\sin \Omega t} \right)\,\frac{\theta }{\,L}.$$

For this objective, it is appropriate to presume the initial condition listed below:

17 $$\theta (0) = 0,\quad {\text{and}}\quad \dot{\theta }(0) = 1.$$

Therefore, the Homotopy equation may be formulated as follows:

18 $$I(\theta ) + \rho \,N(\theta ) = 0;\,\,\rho \in \left[ {0,\,1} \right],$$

where $\rho$ is a synthetic embedded factor. Occasionally, it is known as the Homotopy constraint.

The HPM can offer a variety of approximate solutions, as was clearly demonstrated in our earlier work^26,27^. One of these approaches results in a conventional solution with secular terms; the elimination of these secular terms produces a trivial solution, which is not appropriate. By using the expanded frequency conception, an alternative solution generates a solution that is consistently satisfactory**;** however, it does not satisfy the numerical solution. Therefore, the HPM must consequently be changed once more. In order to investigate the impacts of the delay parameter, which is better at avoiding bifurcations and reducing vibration, we may therefore re-analyze the basic Homotopy equation using a novel expansion in replacement of the conventional expansion. In light of our previous work^23^, we believe that $\theta \left( {t,\rho } \right)$ needs to be further developed. The following are the procedures to obtain the required solution:

Throughout this process^28^, the time-dependent parameter may be formulated as:$\theta (t) = \theta (t;\rho )$ and

19 $$\theta (t;\rho ) = e^{ - \delta \rho t/2} \left( {\theta_{0} (t) + \rho \,\theta_{1} (t) + \cdots } \right).$$

As earlier stated, the homotopy formula for the equation under consideration is provided by Eq. (18). The expanded frequency analysis will serve as the foundation for the instability analysis. This method necessitates the following equation, see Moatimid^26,27^:

20 $$\sigma^{2} = \omega^{2} + \sum\limits_{j = 1}^{\infty } {\rho^{j} \sigma_{j} } .$$

where the factors $\sigma_{i}$ will be calculated later on after being combined with the initial features of the problem. By doing so, secular terms will be made meaningless.

When Eqs. (18–20) are combined, the Laplace transform is applied, the initial conditions from Eq. (17) are considered, and the outcome then becomes:

21 $$L_{T} \left\{ {\theta (t;\,\rho )} \right\} = \frac{1}{{s^{2} + \sigma^{2} }}\, - \frac{\rho }{{s^{2} + \sigma^{2} }}L_{T} \left\{ \begin{gathered} - \sigma_{1} \theta_{0} - \delta \,\dot{\theta }_{0} - \frac{\delta }{2}t\,\ddot{\theta }_{0} - \frac{\delta }{2}t\sigma^{2} \,\theta_{0} + \delta \,\dot{\theta }_{0} - \frac{\Omega \,H}{L}\,\sin \Omega t\,\,\left( {1 - \frac{{\theta_{0}^{2} }}{2}} \right) \hfill \\ - \left( { - 1 + \Omega^{2} \cos \Omega t + \delta \,\Omega \,\sin \Omega t} \right)\,\frac{{\theta_{0}^{3} }}{6\,L} + \left( {\Omega^{2} \cos \Omega t + \delta \,\Omega \,\sin \Omega t} \right)\,\frac{{\theta_{0} }}{\,L} \hfill \\ \end{gathered} \right\}.$$

Findings are obtained by employing the inverse transform to both sides of Eq. (21).

22 $$\theta (t;\,\rho ) = \frac{1}{\sigma }\sin (\sigma \,t) - L_{T}^{ - 1} \left\{ {\frac{\rho }{{s^{2} + \sigma^{2} }}L_{T} \left\{ \begin{gathered} - \sigma_{1} \theta_{0} - \delta \,\dot{\theta }_{0} - \frac{\delta }{2}t\,\ddot{\theta }_{0} - \frac{\delta }{2}t\sigma^{2} \,\theta_{0} + \delta \,\dot{\theta }_{0} - \frac{\Omega \,H}{L}\,\sin \Omega t\,\,\left( {1 - \frac{{\theta_{0}^{2} }}{2}} \right) \hfill \\ - \left( { - 1 + \Omega^{2} \cos \Omega t + \delta \,\Omega \,\sin \Omega t} \right)\,\frac{{\theta_{0}^{3} }}{6\,L} + \left( {\Omega^{2} \cos \Omega t + \delta \,\Omega \,\sin \Omega t} \right)\,\frac{{\theta_{0} }}{\,L}. \hfill \\ \end{gathered} \right\}} \right\}.$$

By producing the development of the dependent variable $\theta (t;\rho )$ as presented in Eq. (22), and then recognizing the coefficients of comparable powers $\rho$ on each side, one obtains

23 $$\rho^{0} :\,\,\theta_{0} (t) = \frac{1}{\sigma }\sin \sigma \,t,$$

and

24 $$\rho :\,\,\,\,\theta_{1} (t) = - L_{T}^{ - 1} \left\{ {\frac{1}{{s^{2} + \sigma^{2} }}L_{T} \left\{ \begin{gathered} - \sigma_{1} \theta_{0} - \delta \,\dot{\theta }_{0} - \frac{\delta }{2}t\,\ddot{\theta }_{0} - \frac{\delta }{2}t\sigma^{2} \,\theta_{0} + \delta \,\dot{\theta }_{0} - \frac{\Omega \,H}{L}\,\sin \Omega t\,\,\left( {1 - \frac{{\theta_{0}^{2} }}{2}} \right) \hfill \\ - \left( { - 1 + \Omega^{2} \cos \Omega t + \delta \,\Omega \,\sin \Omega t} \right)\,\frac{{\theta_{0}^{3} }}{6\,L} + \left( {\Omega^{2} \cos \Omega t + \delta \,\Omega \,\sin \Omega t} \right)\,\frac{{\theta_{0} }}{\,L}. \hfill \\ \end{gathered} \right\}} \right\}.$$

Characteristically, the consistent logical statement goes out from the removal of the secular terms. For this purpose, the coefficient of the function $\sin \sigma \,t$ should be disregarded. This implementation goes to create the parameter $\sigma_{1}$ as follows:

25 $$\sigma_{1} = \frac{1}{{8L\,\sigma^{2} }}.$$

The periodical response at this point in time is provided by

26 $$\begin{aligned} \theta_{1} (t) & = \theta_{11} \cos \,\sigma \,t + \theta_{12} \,\cos \,(\sigma - \Omega )\,t + \theta_{13} \,\cos \,(\sigma + \Omega )\,t + \theta_{14} \,\,\cos \,(3\,\sigma - \Omega )\,t + \theta_{15} \,\cos \,(3\,\sigma + \Omega )\,t \\ & \quad + \theta_{16} \,\sin \,\Omega \,t + \theta_{17} \sin \,\sigma \,t + \theta_{18} \,\sin \,3\,\sigma \,t + \theta_{19} \,\sin \,(\sigma - \Omega )\,t + \theta_{20} \,\sin \,(\sigma + \Omega )\,t + \theta_{21} \,\sin \,(2\,\sigma - \Omega )\,t \\ & \quad + \theta_{22} \,\sin \,(2\,\sigma + \Omega )\,t + \theta_{23} \,\sin \,(3\,\sigma - \Omega )\,t + \theta_{24} \,\sin \,(3\,\sigma + \Omega )\,t, \\ \end{aligned}$$

where $\theta_{ij}$ are given in the Appendix to follow the paper easily.

Consequently, the circumscribed approximate solution of the equation of motion provided in Eq. (14) may be expressed as follows:

27 $$\theta (t) = \mathop {\lim }\limits_{\rho \to 1} e^{ - \delta \rho t/2} \left( {\theta_{0} (t) + \rho \,\theta_{1} (t) + \cdots } \right).$$

In reality, the constrained approximate solution as provided in Eq. (27) requires that the arguments of the trigonometric functions should be of real significance. For this objective, Eq. (25) is inserted into Eq. (20), and it follows that the characteristic nonlinear frequency fulfills a particular equation. To this approximation, the computation indicate**s** that this equation exemplifies a polynomial of fourth degree in the nonlinear frequency. This equation may be written as:

28 $$\sigma^{4} - \omega^{2} \,\sigma^{2} - \frac{1}{8L} = 0.$$

It is convenient to match this procedure with the numerical approach that is identified as RK-4 to evaluate the practicality of the previously expanded frequency implications. The requirements for implementation are listed below. Consequently, in what follows, the analytic approximate solution as provided by Eq. (28) is drawn in blue. Additionally, the RK-4 of the considered structure as offered by Eq. (14) is highlighted in red. The following figure is a graph of a scheme receiving the following specifics:

$$k = 0.9,\,\,L = 0.5,\,\,,\,H = 0.2,\,\,\Omega = 0.01\,\,\,{\text{and}}\,\,\,\delta = 0.1.$$

The computations demonstrate that the synthetic frequency has the amount $\sigma = 1.32038$ and other roots (two are complex conjugate and the third is negative. It is also helpful to compare the numerical solution of the original Eq. (14) generated by RK-4 with the corresponding linear ODE solution. Equation (27) provides the approximate analytical solution. In this comparison, the structure is displayed, as seen in Fig. 2. For an appropriate sample with the provided details, the two figures are obtained in light of the previous data. The findings are reasonably reliable with one another, as can be observed. Further, the mathematical software demonstrated that the absolute difference between the analytical and numerical results, up to a time of 100 units, is 0.186645.

## Linearized stability of the autonomous equation

For more convenience, the linearized stability of the IP may be derived in the special case, in the absence of the periodicity of the pin motion ($\Omega = 0$). In this situation, the governing equation of motion then becomes

29 $$\ddot{\theta } + \delta \,\dot{\theta } + \omega^{2} \,\theta + \frac{1}{6\,L}\,\theta^{3} = 0.$$

Contemplating the conversion: $\dot{\theta } = \phi$, it follows that Eq. (29) may be transformed to the following equation

30 $$\dot{\theta } = g(\theta ,\phi ),\quad \dot{\phi } = h(\theta ,\phi ),$$

where

31 $$g(\theta ,\phi ) = \phi ,\quad \dot{\phi } = h(\theta ,\phi ) = - \delta \,\phi - \omega^{2} \,\theta - \frac{1}{6\,L}\,\theta^{3} .$$

The fixed points (equilibrium points) occur at the points $(\theta_{0} ,\phi_{0} )$,where

32 $$g(\theta_{0} ,\phi_{0} ) = 0,\quad h(\theta_{0} ,\phi_{0} ) = 0.$$

It follows that

33 $$\phi_{0} = 0,$$

and

34 $$\delta \,\phi_{0} + \omega^{2} \,\theta_{0} + \frac{1}{6\,L}\,\theta_{0}^{3} = 0.$$

Consequently, the only fixed point is $(0,0)$. Here, the Jacobian matrix is defined as

35 $$J = \left( {\begin{array}{*{20}c} 0 & 1 \\ { - \omega^{2} - \frac{1}{2\,L}\,\theta_{0}^{2} } & { - \delta } \\ \end{array} } \right).$$

The eigenvalues $\lambda_{r}$, ($r = 1,\,2$) are given by $J - \lambda_{r} \,I_{2 \times 2} = 0$. Therefore, these eigenvalues are provided by

36 $$\lambda_{1,2} = \frac{1}{2}\left( { - \delta \pm \sqrt {\delta^{2} - 4\,\left( {\omega^{2} + \frac{1}{2\,L}\,\theta_{0}^{2} } \right)} \,} \right).$$

When the Jacobian eigenvalues have a negative real portion, the equilibrium point is typically transformed into a stable state. On the contrary, if at least one of the eigenvalues has a positive real portion, the equilibrium point is unstable. As demonstrated by He et al.^17^ and Ghaleb et al.^29^, it is more acceptable to consider a sample system to indicate the stability/instability arrangement in light of the equilibrium points. As a result, the condition is determined by the type of the eigenvalues. Table 1 below provides a summary of this process.

**Table 1:** Equilibria classification of the eigenvalues and their stability/instability.

Values of parameters	Eigenvalues	Classification of the critical point
$\delta = 0$, $L = 0.5$, $k = 0.9$	$\lambda_{1,2} = \pm \,i\,{1}{{.26491 }}$	A stable center. See Fig. 3
$\delta = 0.8$, $L = 0.5$, $k = 0.6$	$\lambda_{1,2} = - \,0.4 \pm \,i\,{0}{{.4899}}$	A stable spiral. See Fig. 4
$\delta = 0.8$, $L = 0.5$, $k = 0.9$	$\lambda_{1,2} = - \,0.4 \pm \,i\,{1}{{.2 }}$	A stable spiral. See Fig. 5
$\delta = 0.5$, $L = 0.5$, $k = 0.9$	$\lambda_{1} = - 4.6564$,$\lambda_{2} = - \,0.3436$	A stable proper node. See Fig. 6
$\delta = - 0.8$, $L = 0.5$, $k = 0.9$	$\lambda_{1,2} = 0.4 \pm \,i\,{1}{{.2 }}$	An unstable spiral. See Fig. 7
$\delta = - \,0.5$, $L = 0.5$, $k = 0.9$	$\lambda_{1} = {4}{{.6564}}$, $\lambda_{2} = 0.3436$	An unstable proper node. See Fig. 8

**Figure 3:**
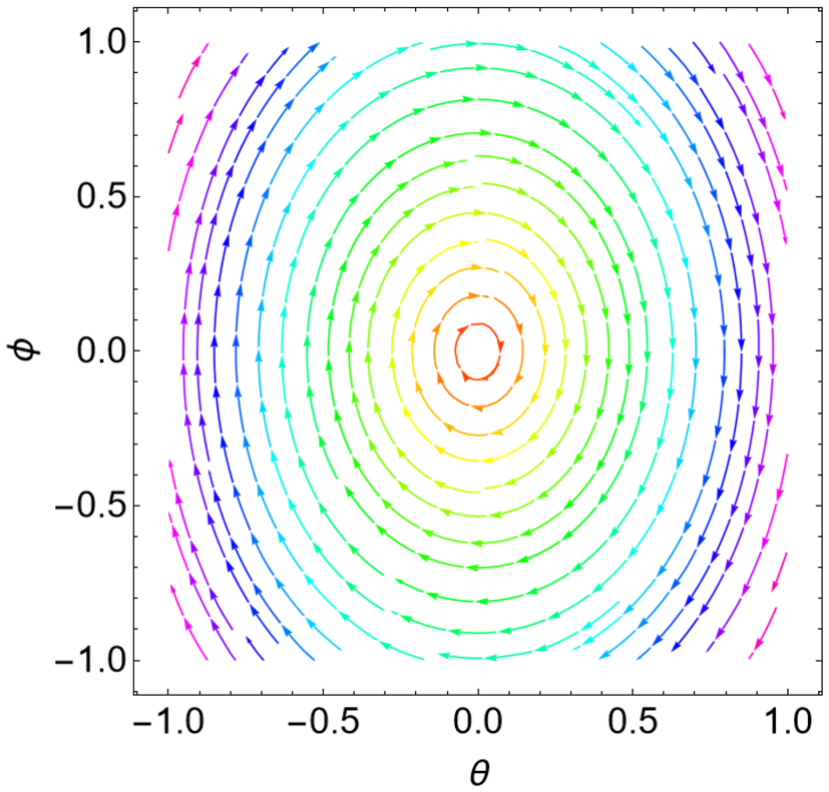
Dynamical performance (stable center) with certain factors recorded in Table 1.

**Figure 4:**
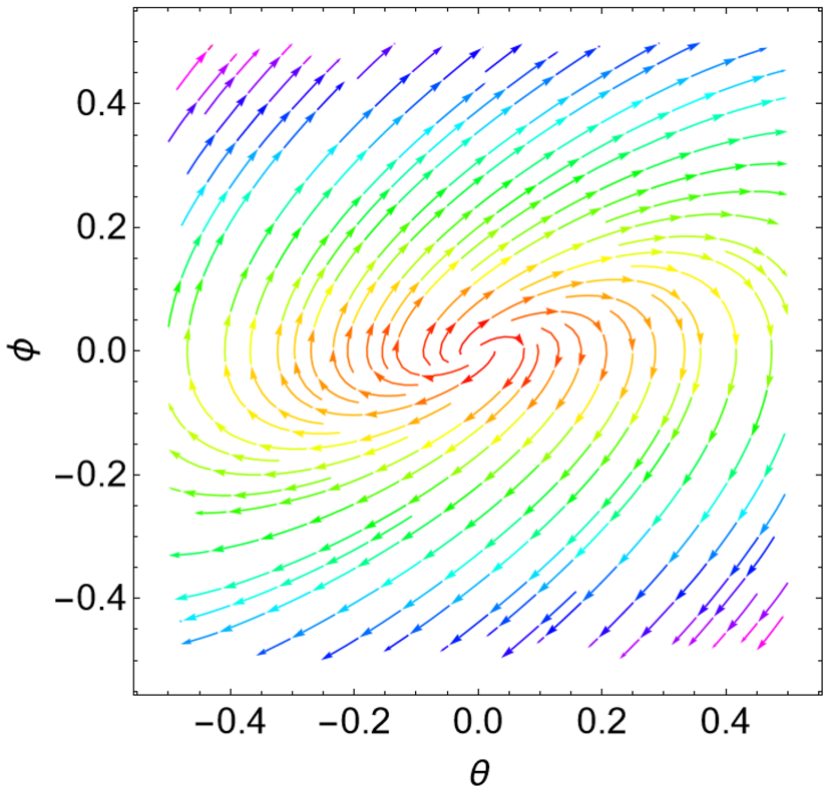
Dynamical performance (stable spiral) with certain factors recorded in Table 1.

**Figure 5:**
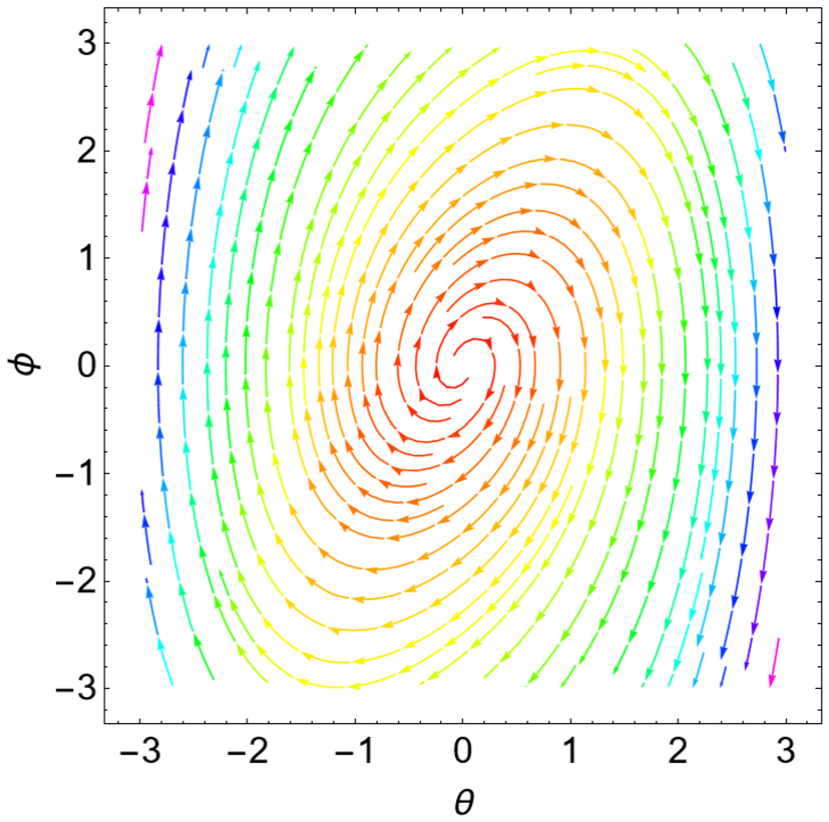
Dynamical behavior (stable spiral) with given factors recorded in Table 1.

**Figure 6:**
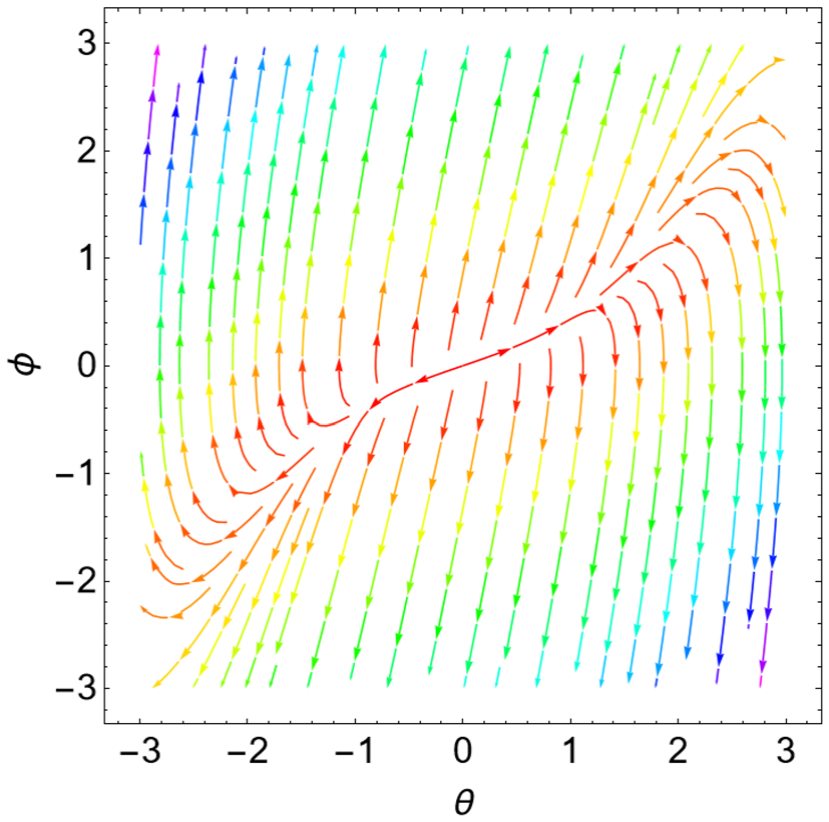
Dynamical performance (stable proper node) with certain parameters listed in Table 1.

**Figure 7:**
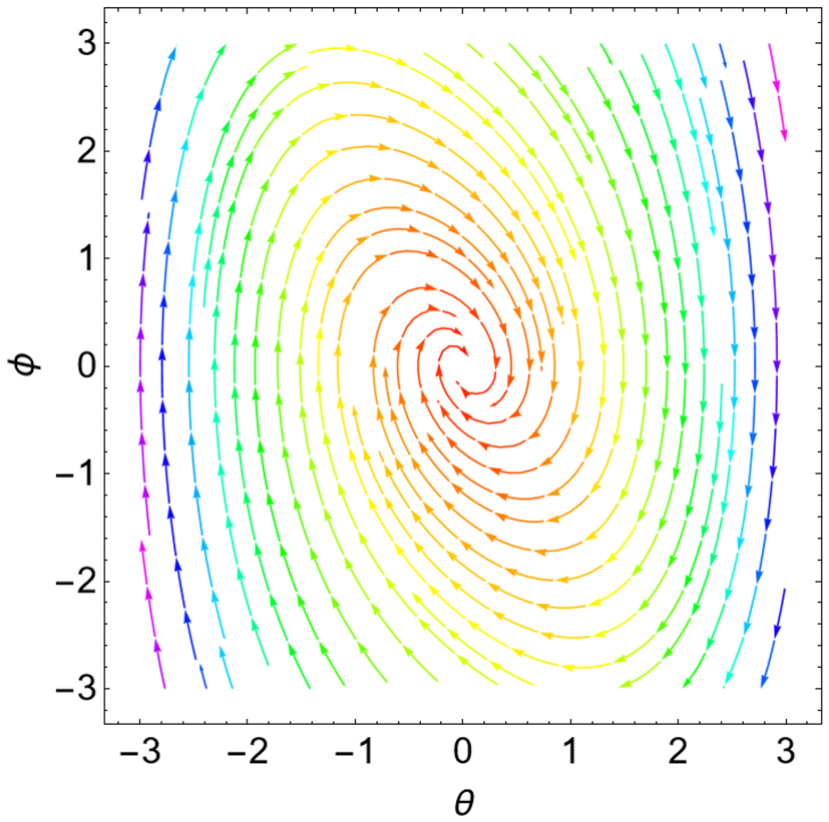
Dynamical performance (unstable spiral) with certain factors recorded in Table 1.

**Figure 8:**
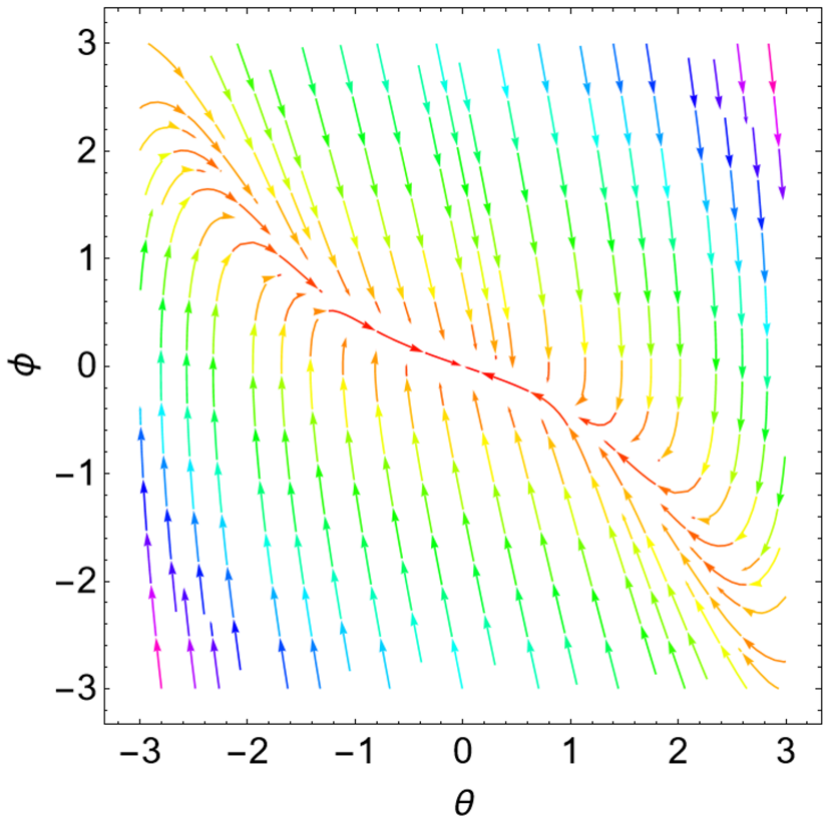
Dynamical performance (unstable proper node) with specified factors recorded in Table 1.

## IP system with PPF controller

Returning again to the fundamental equation of motion as given in (14) and letting $F_{1} = H\,\Omega \,/L$,$F_{2} = \Omega^{2} /L$,$\,F_{3} = \delta \,\Omega \,/L$,$\,\beta = 1/6\,L$, one gets

37 $$\,\ddot{\theta } + \delta \,\dot{\theta } + \omega^{2} \,\theta + \beta \,\theta^{3} = F_{1} \,\,\sin \Omega \,t\,\left( {1 - \theta^{2} /2} \right) - \left( {\,F_{2} \cos \Omega \,t + F_{3} \sin \Omega \,t} \right)\,\left( {\theta - \theta^{3} /6} \right).$$

Adding the PPF controller as previously shown^19–21^ to the preceding equation of motion, the following equations are established as follows:

38 $$\,\ddot{\theta } + \varepsilon \,\tilde{\delta }\,\dot{\theta } + \omega^{2} \,\theta + \varepsilon^{ - 1} \tilde{\beta }\,\theta^{3} = \varepsilon^{2} \,\tilde{F}_{1} \,\,\sin \Omega t\,\left( {1 - \theta^{2} /2} \right) - \varepsilon \left( {\,\tilde{F}_{2} \cos \Omega t + \tilde{F}_{3} \sin \Omega t} \right)\,\left( {\theta - \theta^{3} /6} \right)\, + \varepsilon \,\tilde{q}_{1} \,v\,,$$

and

39 $$\,\ddot{v} + \varepsilon \,\tilde{\delta }_{1} \,\dot{v} + \omega_{1}^{2} \,v = \varepsilon \,\tilde{q}_{2} \,\theta \,.$$

where the coefficients scale are as follows: $\delta = \varepsilon \,\tilde{\delta },\beta = \varepsilon^{ - 1} \tilde{\beta },F_{1} = \varepsilon^{2} \,\tilde{F}_{1} ,F_{2} = \varepsilon \,\tilde{F}_{2} ,F_{3} = \varepsilon \,\tilde{F}_{3} ,q_{1} = \varepsilon \,\tilde{q}_{1}$, $\delta_{1} = \varepsilon \,\tilde{\delta }_{1} \,,{\text{and}}\,\,q_{2} = \varepsilon \,\tilde{q}_{2}$.

$\theta$ and $v$ are the amplitudes of the model of the IP and controller, respectively. $\delta$ and $\delta_{1}$ are the coefficients of damping, $\omega$ and $\omega_{1}$ are natural frequencies, $\beta$ is the coefficients of the nonlinear parameter, $\Omega$ is the excitation frequency, $F_{1}$, $F_{2}$ and $F_{3}$ are the parametric excitations, $q_{1}$ and $q_{2}$ are the parameters of control signals sign.

### Numerical simulation with time history

In what follows, the numerical RK-4 is employed to graph the time history curve as well as the phase portrait before and after combining the PPF control at the primary resonance ($\Omega \cong \omega$) and the internal resonance ($\omega_{1} \cong \omega$). These calculations are performed based on MATLAB® computer program control. For this purpose, the sample chosen system is taken as follows:

$$\delta = 0.02,\,\,\omega = 1.0,\beta = 0.3,F_{1} = 0.5,F_{2} = 0.1,F_{3} = 0.1,q_{1} = 0.2,\delta_{1} = 0.02,q_{2} = 0.5.$$

**Figure 9:**
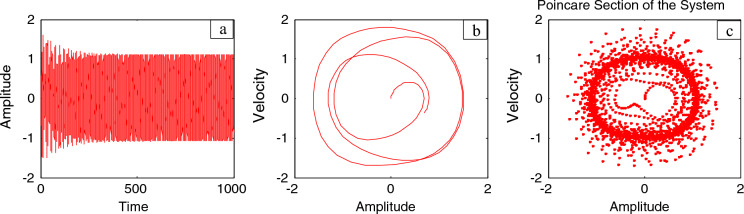
Time history, phase portrait and Poincare map of style without controller at $\Omega = \omega$.

**Figure 10:**
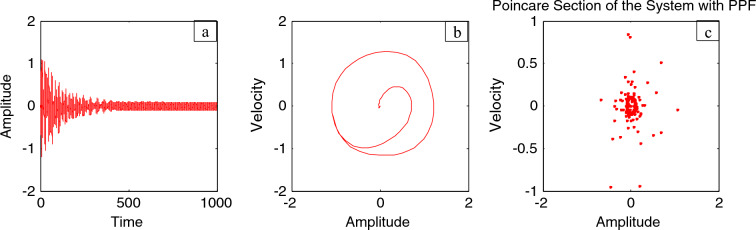
Time history, phase portrait and Poincare map of controlled style at $\Omega = \omega$ and $\omega_{1} = \omega$ .

The time history is illustrated through Fig. 9a for the steady state amplitude of IP system before adding the control. As shown from this figure, the amplitude reaches 1.074. Simultaneously, Fig. 9b represents the phase portrait between the velocity and amplitude for the same case, which shows the chaotic attractor and approximately multi-limit cycle. In addition, the response of the IP with and without the PPF controller is depicted as a Poincare map diagram in Fig. 9c. This figure is depicted to mention the type of motion of the system and the controller. On the other hand, Fig. 10a depicts the amplitude of the considered structure after combining the PPF control. It is found that that amplitude becomes 0.103. Therefore, according to this controller, the amplitudes have been reduced by the ratio 90.5%. Additionally, Fig. 10b displays the phase portrait between the velocity and amplitude after adding the PPF controller, which shows improvement of the chaotic attractor and limited cycle numbers. Finally, the effectiveness of the PPF controller $E_{a}$ is defined as ($E_{a} =$ steady-state amplitude of the structure before PPF separated by after controlling) and is of 10.43. Similar results were obtained earlier in our previous work^19^. As previously shown, Fig. 9c introduced Poincaré map for detecting chaos behavior in IP system before adding PPF controller. Furthermore, Fig. 10c presented the Poincaré map to show that the IP system after applying the PPF controller is no longer chaotic, which indicates a better quality of this controller.

### Perturbation analysis with MSPT

The MSPT^19–21,25,30–32^ is applied to obtain an approximate solution of Eqs. (38) and (39) as follows:

40 $$\theta (t;\varepsilon ) = \varepsilon \,\theta_{0} (T_{0} ,T_{1} ,T_{2} ) + \varepsilon^{2} \,\theta_{1} (T_{0} ,T_{1} ,T_{2} ) + \varepsilon^{3} \,\theta_{2} (T_{0} ,T_{1} ,T_{2} ) + O(\varepsilon^{4} ),$$

and

41 $$v(t;\varepsilon ) = \varepsilon \,v_{0} (T_{0} ,T_{1} ,T_{2} ) + \varepsilon^{2} \,v_{1} (T_{0} ,T_{1} ,T_{2} ) + \varepsilon^{3} \,v_{2} (T_{0} ,T_{1} ,T_{2} ) + O(\varepsilon^{4} ).$$

The time derivatives may be transformed as follows:

42 $$\frac{d}{d\,t} \equiv D_{0} + \varepsilon \,D_{1} + \varepsilon^{2} D_{2} ,$$

and

43 $$\frac{{d^{2} }}{{d\,t^{2} }} \equiv D_{0}^{2} + 2\,\varepsilon \,D_{0} D_{1} + \varepsilon^{2} (D_{1}^{2} + 2D_{0} D_{2} ),$$

where $$T_{k} = \varepsilon^{k} \,t$$ and $$D_{k} \equiv \frac{\partial }{{\partial T_{k} }}$$, ($$k = 0,1,2$$).

Substituting from Eqs. (40)–(43) into Eqs. (38) and (39), and then equating the similar powers for $\varepsilon$ in the two sides, one obtains the following equations:

Order ($$\varepsilon$$):

44 $$(D_{0}^{2} + \omega^{2} )\,\theta_{0} = 0,$$

and

45 $$(D_{0}^{2} + \omega_{1}^{2} )\,v_{0} = 0.$$

Order ($$\varepsilon^{2}$$):

46 $$\begin{aligned} (D_{0}^{2} + \omega^{2} )\,\theta_{1} & = i\,\tilde{F}_{1} (e^{{ - i\Omega T_{0} }} - e^{{i\Omega T_{0} }} )/2 - \tilde{F}_{2} \,\theta_{0} (e^{{ - i\Omega T_{0} }} + e^{{i\Omega T_{0} }} )/2 - i\,\tilde{F}_{3} \,\theta_{0} (e^{{ - i\Omega T_{0} }} - e^{{i\Omega T_{0} }} )/2 \\ & \,\,\,\,\,\, + \tilde{q}_{1} \,v_{0} - \tilde{\beta }\,\theta_{0}^{3} - \tilde{\delta }\,D_{0} \theta_{0} - 2\,D_{0} D_{1} \theta_{0} , \\ \end{aligned}$$

and

47 $$(D_{0}^{2} + \omega_{1}^{2} )\,v_{1} = \tilde{q}_{2} \,\theta_{0} - \tilde{\delta }_{1} \,D_{0} v_{0} - 2\,D_{0} D_{1} v_{0} .$$

Order ($$\varepsilon^{3}$$):

48 $$\begin{aligned} (D_{0}^{2} + \omega^{2} )\,\theta_{2} & = \tilde{q}_{1} \,v_{1} - \tilde{F}_{2} \,\theta_{1} (e^{{ - i\Omega T_{0} }} + e^{{i\Omega T_{0} }} )/2 - i\,\tilde{F}_{3} \,\theta_{1} (e^{{ - i\Omega T_{0} }} + e^{{i\Omega T_{0} }} )/2 - 3\,\tilde{\beta }\,\theta_{0}^{2} \theta_{1} - \tilde{\delta }\,D_{1} \theta_{0} \\ & \,\,\,\,\,\, - \,D_{1}^{2} \theta_{0} - \tilde{\delta }\,D_{0} \theta_{1} - 2\,D_{0} D_{2} \theta_{0} - 2\,D_{0} D_{1} \theta_{1} , \\ \end{aligned}$$

and

49 $$(D_{0}^{2} + \omega_{1}^{2} )\,v_{2} = \tilde{q}_{2} \,\theta_{1} - \tilde{\delta }_{1} \,D_{1} v_{0} - \,D_{1}^{2} v_{0} , - \tilde{\delta }_{1} \,D_{0} v_{1} - 2\,D_{0} D_{2} v_{0} - 2\,D_{0} D_{1} v_{1} .$$

The solutions of Eqs. (44) and (45) may be formulated in the following forms:

50 $$\theta_{0} = A(T_{1} ,T_{2} )\,e^{{i\omega \,T_{0} }} + \overline{A}(T_{1} ,T_{2} )\,e^{{ - i\omega \,T_{0} }} ,$$

and

51 $$v_{0} = B(T_{1} ,T_{2} )\,e^{{i\omega_{1} \,T_{0} }} + \overline{B}(T_{1} ,T_{2} )\,e^{{ - i\omega_{1} \,T_{0} }} .$$

where $A(T_{1} ,T_{2} )$ and $B(T_{1} ,T_{2} )$ are arbitrary complex functions of $T_{1}$, and $T_{2}$.

Substituting Eqs. (50) and (51) into Eqs. (46) and (47), one gets the next equations:

52 $$\begin{aligned} (D_{0}^{2} + \omega^{2} )\,\theta_{1} & = - \,\tilde{\beta }\,A^{3} \,e^{{3i\omega \,\,T_{0} }} + \tilde{q}_{1} \,B\,e^{{i\omega_{1} \,T_{0} }} - i\,\tilde{F}_{1} \,e^{{i\Omega T_{0} }} /2 - (\tilde{F}_{2} \, - i\,\tilde{F}_{3} )\,A\,e^{{i(\omega + \Omega )\,T_{0} }} /2 \\ & \,\,\,\, - (\tilde{F}_{2} \, - i\,\tilde{F}_{3} )\,\overline{A}\,e^{{i(\Omega - \omega )\,T_{0} }} /2 + ( - 3\,\tilde{\beta }\,A^{2} \overline{A} - i\,\tilde{\delta }\,\omega \,A - 2\,i\omega \,D_{1} A\,)\,e^{{i\omega \,\,T_{0} }} + c.c, \\ \end{aligned}$$

and

53 $$(D_{0}^{2} + \omega_{1}^{2} )\,v_{1} = \tilde{q}_{2} \,A\,e^{{i\omega \,T_{0} }} + ( - i\,\tilde{\delta }_{1} \,\omega_{1} B - 2\,i\omega_{1} \,D_{1} B\,)\,e^{{i\omega_{1} T_{0} }} + c.c.$$

where $c.c$ signifies the complex conjugates of the previous terms.

To this end, the purpose of the study is to reduce the vibration of the considered prototype. Consequently, the non-resonance case will be omitted. So, the following investigation focuses only on the resonance cases. It should be noticed that the resonance cases must be determined for Eqs. (52) and (53), namely, the primary resonance ($\Omega = \omega$) and the internal resonance ($\omega_{1} = \omega$). Subsequently, the detuning parameters $\sigma_{1}$ and $\sigma_{2}$ as a closeness to the described frequencies are assumed as follows:

54 $$\Omega = \omega + \sigma_{1} = \omega + \varepsilon \,\tilde{\sigma }_{1} ,\quad \omega_{1} = \omega + \sigma_{2} = \omega + \varepsilon \,\tilde{\sigma }_{2} .$$

Substituting Eq. (54) into Eqs. (52) and (53), the solvability conditions may be formulated as:

55 $$2\,i\omega \,D_{1} A = - i\,\tilde{F}_{1} \,e^{{i\,\,\tilde{\sigma }_{1} \,T_{1} }} /2 + \tilde{q}_{1} \,B\,e^{{i\,\tilde{\sigma }_{2} T_{1} }} - 3\,\tilde{\beta }\,A^{2} \overline{A} - i\,\tilde{\delta }\,\omega \,A,$$

and

56 $$2\,i\omega_{1} \,D_{1} B = \tilde{q}_{2} \,A\,e^{{ - i\,\tilde{\sigma }_{2} \,T_{1} }} - i\,\tilde{\delta }_{1} \,\omega_{1} B.$$

Consequently, the specific solutions of Eqs. (52) and (53), after removing the terms of the secular terms, become

57 $$\theta_{1} = \left[ {\frac{{\tilde{\beta }\,A^{3} }}{{8\,\omega^{2} }}} \right]\,e^{{3i\omega \,\,T_{0} }} + \,\left[ {\frac{{( - \tilde{F}_{2} \, + i\tilde{F}_{3} )\,\overline{A}}}{{2\,\left( {\omega^{2} - \,(\Omega - \omega )^{2} } \right)}}} \right]e^{{i(\Omega - \omega )\,T_{0} }} + \left[ {\frac{{( - \tilde{F}_{2} \, + i\tilde{F}_{3} )A}}{{2\,\left( {\omega^{2} - \,(\Omega + \omega )^{2} } \right)}}} \right]e^{{i(\omega + \Omega )\,T_{0} }} + c.c,$$

and

58 $$v_{1} = 0.$$

Again, Substituting Eqs. (50), (51), (57) and (58) into Eqs. (48) and (49), the second order equations may be reduced to the following equations:

59 $$\begin{aligned} (D_{0}^{2} + \omega^{2} )\,\theta_{2} & = \theta_{25} \,e^{{i\omega T_{0} }} + \theta_{26} \,e^{{3\,i\omega \,\,T_{0} }} + \theta_{27} \,e^{{5i\omega \,\,T_{0} }} + \theta_{28} \,e^{{i(\Omega + \omega )\,\,T_{0} }} + \theta_{29} \,e^{{i(\Omega - \omega )\,\,T_{0} }} \\ & \,\,\,\,\,\, + \theta_{30} \,e^{{i(\Omega + 3\omega )\,\,T_{0} }} + \theta_{31} \,e^{{i(\Omega - 3\omega )\,\,T_{0} }} + \theta_{32} \,e^{{i(2\,\Omega + \omega )\,\,T_{0} }} + \theta_{33} \,e^{{i(2\,\Omega - \omega )\,\,T_{0} }} + c.c, \\ \end{aligned}$$

and

60 $$(D_{0}^{2} + \omega_{1}^{2} )\,v_{2} = \theta_{34} \,e^{{i(\Omega - \omega )\,T_{0} }} + \theta_{35} \,e^{{i(\omega + \Omega )\,T_{0} }} + \theta_{36} \,e^{{3i\omega \,T_{0} }} + \theta_{37} \,e^{{i\omega_{1} T_{0} }} + c.c.$$

where $\theta_{ij}$ are given in the Appendix to follow the paper easily.

Once more, the solvability condition for Eqs. (59) and (60) is

61 $$2i\omega \,D_{2} A = \frac{{ - 3\,\,\tilde{\beta }^{2} \,A^{3} \,\overline{A}^{2} }}{{8\,\omega^{2} }} + \frac{{A\,(\tilde{F}_{2}^{2} \, + \tilde{F}_{3}^{2} )}}{{4\,(\omega^{2} - (\Omega + \omega )^{2} )}} + \frac{{A\,(\tilde{F}_{2}^{2} \, + \tilde{F}_{3}^{2} )}}{{4\,(\omega^{2} - (\Omega - \omega )^{2} )}} - \tilde{\delta }\,D_{1} A - D_{1}^{2} + \frac{{\overline{A}(\tilde{F}_{2} - i\,\tilde{F}_{3} )^{2} }}{{4\,(\omega^{2} - (\Omega - \omega )^{2} )}}\,e^{{2\,i\,\tilde{\sigma }_{1} \,T_{1} }} ,$$

and

62 $$2\,i\omega_{1} \,D_{2} B = - \tilde{\delta }_{1} \,D_{1} B - \,D_{1}^{2} B.$$

Consequently, the solutions of Eqs. (59) and (60) after removing the secular terms are

63 $$\begin{aligned} \theta_{2} & = \frac{{ - \,\theta_{26} }}{{8\,\omega^{2} }}\,e^{{3\,i\omega \,\,T_{0} }} - \frac{{\theta_{27} }}{{24\,\omega^{2} }}\,e^{{5i\omega \,\,T_{0} }} + \frac{{\theta_{28} }}{{(\omega^{2} - (\Omega + \omega )^{2} )}}\,e^{{i(\Omega + \omega )\,\,T_{0} }} + \frac{{\theta_{29} }}{{(\omega^{2} - (\Omega - \omega )^{2} )}}\,e^{{i(\Omega - \omega )\,\,T_{0} }} \\ & \,\,\, + \frac{{\theta_{30} }}{{(\omega^{2} - (\Omega + 3\,\omega )^{2} )}}\,e^{{i(\Omega + 3\omega )\,\,T_{0} }} + \frac{{\theta_{31} }}{{(\omega^{2} - (\Omega - 3\,\omega )^{2} )}}\,e^{{i(\Omega - 3\omega )\,\,T_{0} }} + \frac{{\theta_{32} }}{{(\omega^{2} - (2\,\Omega + \omega )^{2} )}}\,e^{{i(2\,\Omega + \omega )\,\,T_{0} }} + c.c, \\ \end{aligned}$$

and

64 $$v_{2} = \frac{{\theta_{34} }}{{(\omega_{1}^{2} - (\Omega - \omega )^{2} )}}\,e^{{i(\Omega - \omega )\,T_{0} }} + \frac{{\theta_{35} }}{{(\omega_{1}^{2} - (\Omega + \omega )^{2} )}}\,e^{{i(\omega + \Omega )\,T_{0} }} + \frac{{\theta_{34} }}{{(\omega_{1}^{2} - 9\,\omega^{2} )}}\,e^{{3i\omega \,T_{0} }} + c.c.$$

The general approximate solution of Eqs. (38) and (39) can be obtained by exchanging Eqs. (50), (51), (57), (58), (63) and (64) into Eqs. (40)-(41). Additionally, in the next section, we will study the stability of the measured resonance case.

## Linearized stability of the non-autonomous equation

Considering Eq. (42), the combinations of Eqs. (55) and (61), then Eqs. (56) and (62) yield the following equations:

65 $$\begin{aligned} 2\,i\omega \,\frac{dA}{{d\,t}} & = \varepsilon \,\left( { - i\,\tilde{F}_{1} \,e^{{i\,\,\tilde{\sigma }_{1} \,T_{1} }} /2 + \tilde{q}_{1} \,B\,e^{{i\,\tilde{\sigma }_{2} T_{1} }} - 3\,\tilde{\beta }\,A^{2} \overline{A} - i\,\tilde{\delta }\,\omega \,A) + \varepsilon^{2} \,\left\{ {\frac{{ - 3\,\,\tilde{\beta }^{2} \,A^{3} \,\overline{A}^{2} }}{{8\,\omega^{2} }} + \frac{{A\,(\tilde{F}_{2}^{2} \, + \tilde{F}_{3}^{2} )}}{{4\,(\omega^{2} - (\Omega + \omega )^{2} )}}} \right.} \right. \\ & \,\,\,\,\,\,\,\,\, + \left. {\frac{{A\,(\tilde{F}_{2}^{2} \, + \tilde{F}_{3}^{2} )}}{{4\,(\omega^{2} - (\Omega - \omega )^{2} )}}} \right) - \tilde{\delta }\,\left( {\frac{{ - \tilde{F}_{1} \,e^{{i\,\,\tilde{\sigma }_{1} \,T_{1} }} }}{4\,\omega } - \frac{{i\,\tilde{q}_{1} \,B}}{2\,\omega }\,e^{{i\,\tilde{\sigma }_{2} T_{1} }} + \frac{{3\,i\,\tilde{\beta }\,A^{2} \overline{A}}}{2\,\omega } - \frac{{\,\tilde{\delta }\,A}}{2\,}} \right) - \left( {\frac{{\tilde{\delta }^{2} \,A}}{4\,} - \frac{{3\,i\,\tilde{\delta }\,\tilde{\beta }\,A^{2} \,\overline{A}}}{\omega }} \right. \\ & \,\,\,\,\,\,\,\,\, - \frac{{9\,\tilde{\beta }^{2} \,A^{3} \,\overline{A}^{2} }}{{4\,\omega^{2} }} - \frac{{\tilde{q}_{1} \,\tilde{q}_{2} \,A}}{{4\,\omega \,\omega_{1} }} + \left( {\frac{{\tilde{\delta }\,\tilde{F}_{1} }}{8\,\omega } - \frac{{3\,i\,\tilde{\beta }\,\tilde{F}_{1} \,A\,\overline{A}}}{{4\,\omega^{2} }} - \frac{{i\,\tilde{F}_{1} \,\tilde{\sigma }_{1} }}{4\,\omega }} \right)\,e^{{i\,\,\tilde{\sigma }_{1} \,T_{1} }} + \left( {\frac{{i\,\tilde{q}_{1} \,B\,(\tilde{\delta } + \tilde{\delta }_{1} )}}{4\,\omega } + \frac{{3\,\tilde{\beta }\,\tilde{q}_{1} \,B\,A\,\overline{A}}}{{2\,\omega^{2} }}} \right. \\ & \,\,\,\,\,\,\,\,\, + \left. {\left. {\frac{{\tilde{q}_{1} \,\tilde{\sigma }_{2} \,B}}{2\,\omega }} \right)e^{{i\,\tilde{\sigma }_{2} T_{1} }} - \left( {\frac{{3\,i\,\tilde{\beta }\,\tilde{F}_{1} \,A^{2} }}{{8\,\omega^{2} }}} \right)\,e^{{ - i\,\,\tilde{\sigma }_{1} \,T_{1} }} - \left. {\left( {\frac{{3\,\tilde{\beta }\,\tilde{q}_{1} \,\overline{B}\,A^{2} }}{{4\,\omega^{2} }}} \right)\,e^{{ - i\,\,\tilde{\sigma }_{2} \,T_{1} }} } \right) + \frac{{\overline{A}(\tilde{F}_{2} - i\,\tilde{F}_{3} )^{2} }}{{4\,(\omega^{2} - (\Omega - \omega )^{2} )}}\,e^{{2\,i\,\tilde{\sigma }_{1} \,T_{1} }} } \right\}, \\ \end{aligned}$$

and

66 $$\begin{aligned} 2\,i\omega_{1} \,\frac{dB}{{d\,t}} & = \varepsilon \left( {\tilde{q}_{2} \,A\,e^{{ - i\,\,\sigma_{2} \,T_{1} }} - i\,\tilde{\delta }_{1} \,\omega_{1} B) + \,\varepsilon^{2} \left\{ { - \tilde{\delta }_{1} \,\left( {\frac{{ - i\,\tilde{q}_{2} \,A}}{{2\,\omega_{1} }}\,e^{{ - i\,\tilde{\sigma }_{2} \,T_{1} }} - \frac{{\tilde{\delta }_{1} \,}}{2\,}B} \right) - \left( {\frac{{\tilde{\delta }_{1}^{2} \,B}}{4} - \frac{{\tilde{q}_{1} \,\tilde{q}_{2} \,B}}{4\,\omega \,\omega }} \right.} \right.} \right. \\ & \,\,\,\,\, + \left. {\left. {\left( {\frac{{i\,\tilde{q}_{2} \,(\tilde{\delta } + \tilde{\delta }_{1} )\,A}}{{4\,\omega_{1} }} + \frac{{3\,\tilde{q}_{2} \,\tilde{\beta }\,A^{2} \,\overline{A}}}{{4\,\omega \,\omega_{1} }} - \frac{{\tilde{\sigma }_{2} \,\tilde{q}_{2} \,A}}{{2\,\omega_{1} }}} \right)\,e^{{ - i\,\tilde{\sigma }_{2} \,T_{1} }} + \frac{{i\,\tilde{q}_{2} \,\tilde{F}_{1} }}{{8\,\omega \,\omega_{1} }}e^{{i\,(\tilde{\sigma }_{1} - \tilde{\sigma }_{2} )\,T_{1} }} } \right)} \right\}. \\ \end{aligned}$$

The coupled system of Eqs. (65) and (66) is first order nonlinear ordinary differential equations of $A$ and $B$ of complex coefficients. To study the solution of Eqs. (65) and (66), it is appropriate to use the polar form for the complex functions $A(T_{1} ,T_{2} )$ and $B(T_{1} ,T_{2} )$ as:

67 $$A = \frac{{\tilde{a}_{1} }}{2}e^{{i\rho_{1} }} \quad {\text{and}}\quad B = \frac{{\tilde{a}_{2} }}{2}e^{{i\rho_{2} }} ,$$

where $\,\tilde{a}_{1} ,\,\,\,\tilde{a}_{2} ,\,\,\rho_{1}$ and $\,\,\rho_{2}$ are real functions on the time $\,\,t$.

We may investigate these functions as $a_{1} = \varepsilon \,\tilde{a}_{1}$, $a_{2} = \varepsilon \,\tilde{a}_{2}$.The direct differentiation of the previous functions provides

68 $$\dot{A} = \frac{{\dot{\tilde{a}}_{1} }}{2}e^{{i\rho_{1} }} + i\frac{{\tilde{a}_{1} }}{2}\dot{\rho }_{1} e^{{i\rho_{1} }} \quad {\text{and}}\quad \dot{B} = \frac{{\dot{\tilde{a}}_{2} }}{2}e^{{i\rho_{2} }} + i\frac{{\tilde{a}_{2} }}{2}\dot{\rho }_{2} e^{{i\rho_{2} }} .$$

Using Eqs. (67) and (68) into Eqs. (65) and (66), with the restoration of each scaled factor to its basic form, then distinguishing the real and imaginary elements yield the following

69 $$\begin{aligned} \dot{a}_{1} & = - \frac{\delta }{2}a_{1} + \frac{3\beta \,\delta }{{16\,\omega^{2} }}a_{1}^{3} + \frac{{F_{1} \,(9\beta \,a_{1}^{2} + 8\,\omega \,(\sigma_{1} - 2\omega ))}}{{32\,\omega^{3} }}\cos \psi_{1} + \frac{{\,\delta \,F_{1} }}{{8\,\omega^{2} }}\,\sin \psi_{1} + \frac{{q_{1} \,(\delta - \,\delta_{1} )}}{{8\,\omega^{2} }}a_{2} \,\cos \psi_{2} \\ & \,\,\,\,\, + \frac{{q_{1} \,( - 9\beta \,a_{1}^{2} + 8\,\omega \,( - \sigma_{2} + 2\omega ))}}{{32\,\omega^{3} }}\,a_{2} \,\sin \psi_{2} - \frac{{F_{2} \,F_{3} }}{4\,\omega \,\Omega \,(2\,\omega - \Omega )}\,a_{1} \,\cos 2\,\psi_{1} + \frac{{(F_{2}^{2} - F_{3}^{2} )}}{8\,\omega \,\Omega \,(2\,\omega - \Omega )}\,a_{1} \,\sin 2\,\psi_{1} , \\ \end{aligned}$$

70 $$\begin{aligned} a_{1} \,\dot{\rho }_{1} & = - \frac{{\delta^{2} }}{8\,\omega }\,a_{1} - \frac{{q_{1} \,q_{2} }}{{8\,\omega_{1} \,\omega^{2} }}\,a_{1} + \frac{3\,\beta }{{8\,\omega }}\,a_{1}^{3} - \frac{{15\,\beta^{2} }}{{256\,\omega^{3} }}\,a_{1}^{5} - \frac{{(\,F_{2}^{2} + F_{3}^{2} )}}{4\,\omega \,(2\,\omega - \Omega )\,(2\,\omega + \Omega )}\,a_{1} - \frac{{\delta \,F_{1} }}{{8\,\omega^{2} }}\,\cos \psi_{1} \\ & \,\,\,\,\,\,\,\,\,\, + \frac{{F_{1} \,(3\beta \,a_{1}^{2} + 8\,\omega \,(\sigma_{1} - 2\omega ))}}{{32\,\omega^{3} }}\sin \psi_{1} - \frac{{(F_{2}^{2} \, - F_{3}^{2} )}}{8\,\omega \,\Omega \,(2\,\omega - \Omega )}\,a_{1} \,\cos 2\,\psi_{1} - \frac{{F_{2} \,F_{3} }}{4\,\omega \,\Omega \,(2\,\omega - \Omega )}\,a_{1} \,\sin 2\psi_{1} \\ & \,\,\,\,\,\,\,\,\,\, + \frac{{q_{1} \,(\delta - \,\delta_{1} )}}{{8\,\omega^{2} }}a_{2} \,\sin \psi_{2} + \frac{{q_{1} \,(3\beta \,a_{1}^{2} + 8\,\omega \,(\sigma_{2} - 2\omega ))}}{{32\,\omega^{3} }}\,a_{2} \,\cos \psi_{2} , \\ \end{aligned}$$

71 $$\dot{a}_{2} = - \frac{1}{2}\,\delta_{1} \,a_{2} - \frac{{q_{2} (\delta - \,\delta_{1} )}}{{8\,\omega_{1}^{2} }}\,a_{1} \,\cos \psi_{2} + \frac{{q_{2} (3\beta \,a_{1}^{2} - 8\omega \,\sigma_{2} - 16\,\omega \,\omega_{1} )}}{{32\,\omega \,\omega_{1}^{2} }}\,a_{1} \,\sin \psi_{2} - \frac{{F_{1} \,q_{2} }}{{8\,\omega \,\omega_{1}^{2} }}\,\cos (\psi_{1} - \psi_{2} ),$$

72 $$\begin{aligned} a_{2} \,\dot{\rho }_{2} & = - \frac{{q_{1} \,q_{2} }}{{8\,\omega \,\omega_{1}^{2} }}\,a_{2} - \frac{{\delta_{1}^{2} }}{{8\,\omega_{1} }}\,a_{2} + \frac{{q_{2} (\delta - \,\delta_{1} )}}{{8\,\omega_{1}^{2} }}\,a_{1} \,\sin \psi_{2} + \frac{{q_{2} (3\,\beta \,a_{1}^{2} - 8\,\omega \,\sigma_{2} - 16\,\omega \,\omega_{1} )}}{{32\,\omega \,\omega_{1}^{2} }}\,a_{1} \,\cos \psi_{2} \\ & \,\,\,\,\,\,\,\,\,\,\,\, - \frac{{F_{1} \,q_{2} }}{{8\,\omega \,\omega_{1}^{2} }}\,\sin (\psi_{1} - \psi_{2} ). \\ \end{aligned}$$

where $\psi_{1} = \sigma_{1} t - \rho_{1}$ and $\psi_{2} = \sigma_{2} t - \rho_{1} + \rho_{2}$, therefore $\dot{\psi }_{1} = \sigma_{1} - \dot{\rho }_{1}$ and $\dot{\psi }_{2} = \sigma_{2} - \dot{\rho }_{1} + \dot{\rho }_{2} = \sigma_{2} - \sigma_{1} + \dot{\psi }_{1} + \dot{\rho }_{2}$, one finds

73 $$\begin{aligned} \dot{\psi }_{1} & = \sigma_{1} + \frac{{\delta^{2} }}{8\,\omega } + \frac{{q_{1} \,q_{2} }}{{8\,\omega_{1} \,\omega^{2} }} - \frac{3\,\beta }{{8\,\omega }}\,a_{1}^{2} + \frac{{15\,\beta^{2} }}{{256\,\omega^{3} }}\,a_{1}^{4} + \frac{{(\,F_{2}^{2} + F_{3}^{2} )}}{4\,\omega \,(2\,\omega - \Omega )\,(2\,\omega + \Omega )} \\ &\quad- \frac{{F_{1} \,(3\beta \,a_{1}^{2} + 8\,\omega \,(\sigma_{1} - 2\omega ))}}{{32\,\omega^{3} \,a_{1} }}\sin \psi_{1} + \frac{{\delta \,F_{1} }}{{8\,\omega^{2} \,a_{1} }}\,\cos \psi_{1} \\ &\quad + \frac{{(F_{2}^{2} \, - F_{3}^{2} )}}{8\,\omega \,\Omega \,(2\,\omega - \Omega )}\,\cos 2\psi_{1} + \frac{{F_{2} \,F_{3} }}{4\,\omega \,\Omega \,(2\,\omega - \Omega )}\,\sin 2\psi_{1} \\ &\quad- \frac{{q_{1} \,(\delta - \,\delta_{1} )}}{{8\,\omega^{2} }}\,\frac{{a_{2} }}{{a_{1} }}\,\sin \psi_{1} \, - \frac{{q_{1} \,(3\beta \,a_{1}^{2} + 8\,\omega \,(\sigma_{2} - 2\omega ))}}{{32\,\omega^{3} }}\,\frac{{a_{2} }}{{a_{1} }}\,\cos \psi_{2} \\ \end{aligned}$$

74 $$\begin{aligned} \dot{\psi }_{2} & = \sigma_{2} + \frac{{\delta^{2} }}{8\,\omega } + \frac{{q_{1} \,q_{2} }}{{8\,\omega_{1} \,\omega^{2} }} - \frac{3\,\beta }{{8\,\omega }}\,a_{1}^{2} + \frac{{15\,\beta^{2} }}{{256\,\omega^{3} }}\,a_{1}^{4} + \frac{{(\,F_{2}^{2} + F_{3}^{2} )}}{4\,\omega \,(2\,\omega - \Omega )\,(2\,\omega + \Omega )} \\ &\quad- \frac{{F_{1} \,(3\beta \,a_{1}^{2} + 8\,\omega \,(\sigma_{1} - 2\omega ))}}{{32\,\omega^{3} \,a_{1} }}\sin \psi_{1} + \frac{{\delta \,F_{1} }}{{8\,\omega^{2} \,a_{1} }}\,\cos \psi_{1} + \frac{{(F_{2}^{2} \, - F_{3}^{2} )}}{8\,\omega \,\Omega \,(2\,\omega - \Omega )}\,\cos 2\,\psi_{1} \\ & \quad + \frac{{F_{2} \,F_{3} }}{4\,\omega \,\Omega \,(2\,\omega - \Omega )}\,\sin 2\,\psi_{1} - \frac{{q_{1} \,(3\beta \,a_{1}^{2} + 8\,\omega \,(\sigma_{2} - 2\omega ))}}{{32\,\omega^{3} }}\,\frac{{a_{2} }}{{a_{1} }}\,\cos \psi_{2} - \frac{{q_{1} \,(\delta - \,\delta_{1} )}}{{8\,\omega^{2} }}\,\frac{{a_{2} }}{{a_{1} }}\,\sin \psi_{2} \\ & \,\,\,\,\,\, - \frac{{q_{1} \,q_{2} }}{{8\,\omega \,\omega_{1}^{2} }} - \frac{{\delta_{1}^{2} }}{{8\,\omega_{1} }} + \frac{{q_{2} (\delta - \,\delta_{1} )}}{{8\,\omega_{1}^{2} }}\,\frac{{a_{1} }}{{a_{2} }}\,\sin \psi_{2} + \frac{{q_{2} (3\,\beta \,a_{1}^{2} - 8\,\omega \,\sigma_{2} - 16\,\omega \,\omega_{1} )}}{{32\,\omega \,\omega_{1}^{2} }}\,\frac{{a_{1} }}{{a_{2} }}\,\cos \psi_{2} \\ & \quad - \frac{{F_{1} \,q_{2} }}{{8\,\omega \,\omega_{1}^{2} \,a_{2} }}\,\sin (\psi_{1} - \psi_{2} ) \\ \end{aligned}$$

Equations (69), (71), (73) and (74) are defined as autonomous amplitude-phase modulating equations. We will use these equations to investigate the linearized stability in case of the presence of periodic fields in the governing equation of IP before adding the controller (i.e. putting $a_{2} = 0$, $\psi_{2} = 0$). The following procedure comes out from He et al.^17^ and Hao et al.^33^. For this purpose, Eqs. (69) and (73) are used and the following assumptions are considered:

75 $$\dot{a}_{1} = f(a_{1} ,\psi_{1} ),\quad a_{1} \,\dot{\psi }_{1} = u(a_{1} ,\psi_{1} ).$$

The critical points are determined from the following equations:

76 $$f(a_{1} ,\psi_{1} ) = 0,\quad u(a_{1} ,\psi_{1} ) = 0,$$

Here, the Jacobian matrix is defined as

77 $$\Gamma = \left( {\begin{array}{*{20}c} {\frac{\partial f}{{\partial a_{1} }}} & {\frac{\partial f}{{\partial \psi_{1} }}} \\ {\frac{\partial u}{{\partial a_{1} }}} & {\frac{\partial u}{{\partial \psi_{1} }}} \\ \end{array} } \right).$$

The eigenvalues $\Lambda_{r}$, ($r = 1,\,2$) are given from the characteristic equation $\Gamma - \Lambda_{r} \,I_{2 \times 2} = 0$. Actually, the use of the Mathematica software 13.0.0.0 yields several characteristic values. They are not included here to keep the paper from getting too long. Additionally, in our earlier work^29^, a characterization of the connection between the nature of these roots and the various forms of stability was provided. Table 2 offers a review of the results of the previous operations with regard to various amounts of the system factors.

**Table 2:** Equilibria classification of the eigenvalues and their stability/instability.

	Critical points ($a_{1_0}$, $\theta_{1_{0}}$ + 6.283 m),$m \in Z$	Eigenvalues $\Lambda_{1,2} = a \pm \,i\,{\text{b }}$	Classification of the critical points
Figure 11 $\sigma_{1} = 0.5$, $\delta = 0.02$, $\omega = 1$, $\beta = 0.3$, $F_{1} = 0.5$, $F_{2} = 0.1$, $F_{3} = 0.1$	(− 0.381, 1.55 + 6.283 m), (− 2.328, 1.64 + 6.283 m), (2.675, 1.61 + 6.283 m), (3.85, − 1.67 + 6.283 m), (− 3.887, − 1.48 + 6.283 m)	$\Lambda_{1} < 0 < \Lambda_{2} \,$	An unstable saddle
	(0.381, − 1.59 + 6.283 m), (2.328, − 1.51 + 6.283 m), (− 2.675, − 1.53 + 6.283 m), (− 3.85, 1.49 + 6.283 m), (3.887, 1.66 + 6.283 m)	$\Lambda_{1,2} = \pm \,i\,{\text{b }}$	A stable center
Figure 12 $\sigma_{1} = 0.5$, $\delta = 0.02$, $\omega = 0.2$, $\beta = 0.3$, $F_{1} = 0.5$, $F_{2} = 0.1$, $F_{3} = 0.1$	(− 1.557, − 1.568 + 6.283 m),	$\Lambda_{1} < 0 < \Lambda_{2} \,$	An unstable saddle
	(1.557,1.573 + 6.283 m),	$\Lambda_{1,2} = a \pm \,i\,{\text{b , a}} > {0}$	An unstable spiral
Figure 13 $\sigma_{1} = 0.5$, $\delta = 0.3$, $\omega = 1$, $\beta = 0.3$, $F_{1} = 0.5$, $F_{2} = 0.1$, $F_{3} = 0.1$	(− 0.356, 1.181 + 6.283 m), (− 2.61, − 3.13 + 6.283 m), (2.737, 1.26 + 6.283 m), (3.829, − 2.55 + 6.283 m), (− 3.863, − 0.47 + 6.283 m)	$\Lambda_{1} < 0 < \Lambda_{2} \,$	An unstable saddle
	(0.356, − 1.96 + 6.283 m),	$\Lambda_{1,2} = a \pm \,i\,{\text{b , a}} < {0}$	A stable spiral
	(2.61, 0.01 + 6.283 m), (− 2.737, − 1.88 + 6.283 m), (− 3.829, 0.59 + 6.283 m), (3.863, 2.67 + 6.283 m),	$\Lambda_{1,2} = a \pm \,i\,{\text{b , a}} > {0}$	An unstable spiral
Figure 14 $\sigma_{1} = 0.5$, $\delta = - \,0.3$, $\omega = 1$, $\beta = 0.3$, $F_{1} = 0.5$, $F_{2} = 0.1$, $F_{3} = 0.1$	(− 0.358, 1.97 + 6.283 m), (− 2.582, 0.19 + 6.283 m), (2.71, 2.07 + 6.283 m), (− 3.847, − 2.73 + 6.283 m), (3.848, − 0.51 + 6.283 m)	$\Lambda_{1} < 0 < \Lambda_{2} \,$	An unstable saddle
	(2.582, − 2.95 + 6.283 m), (− 2.71, − 1.07 + 6.283 m), (3.847, 0.41 + 6.283 m), (− 3.848, 2.63 + 6.283 m),	$\Lambda_{1,2} = a \pm \,i\,{\text{b , a}} < {0}$	A stable spiral
	(0.358, − 1.17 + 6.283 m),	$\Lambda_{1,2} = a \pm \,i\,{\text{b , a}} > {0}$	An unstable spiral
Figure 15 $\sigma_{1} = 0.5$, $\delta = 0.02$, $\omega = 1$, $\beta = 0.3$, $F_{1} = 5$, $F_{2} = 0.1$, $F_{3} = 0.1$	(− 2.097, 0.18 + 6.283 m), (− 2.119 ,2.96 + 6.283 m), (3.325, 1.58 + 6.283 m), (3.766, − 1.58 + 6.283 m), (− 4.157, − 1.56 + 6.283 m)	$\Lambda_{1} < 0 < \Lambda_{2} \,$	An unstable saddle
	(2.097, − 2.96 + 6.283 m)	$\Lambda_{1} < \Lambda_{2} < {0}$	A stable proper node
	(2.119, − 0.19 + 6.283 m)	$\Lambda_{1} . > \Lambda_{2} > {0}$	An unstable proper node
	(− 3.325, − 1.56 + 6.283 m), (− 3.766, 1.57 + 6.283 m), (4.157, 1.58 + 6.283 m)	$\Lambda_{1,2} = \pm \,i\,{\text{b }}$	A stable center
Figure 16 $\sigma_{1} = 0.5$, $\delta = 0.02$, $\omega = 1$, $\beta = 0.3$, $F_{1} = 0.5$, $F_{2} = 0.1$, $F_{3} = 2$	(− 1.08, 1.6 + 6.283 m), (2.801, − 0.41 + 6.283 m), (− 2.901, − 2.84 + 6.283 m)	$\Lambda_{1} < 0 < \Lambda_{2} \,$	An unstable saddle
	(0.108, − 1.54 + 6.283 m)	$\Lambda_{1,2} = a \pm \,i\,{\text{b , a}} > {0}$	An unstable spiral
	(− 2.801, 2.73 + 6.283 m), (2.901, 0.30 + 6.283 m)	$\Lambda_{1} . > \Lambda_{2} > {0}$	An unstable proper node

**Figure 11:**
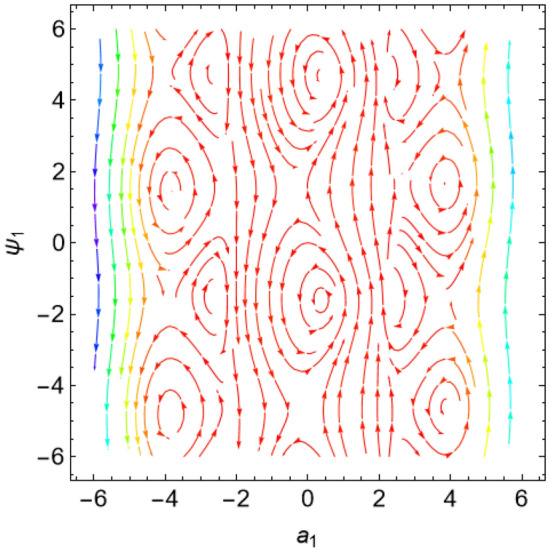
Phase portrait at $\delta = 0.02$.

**Figure 12:**
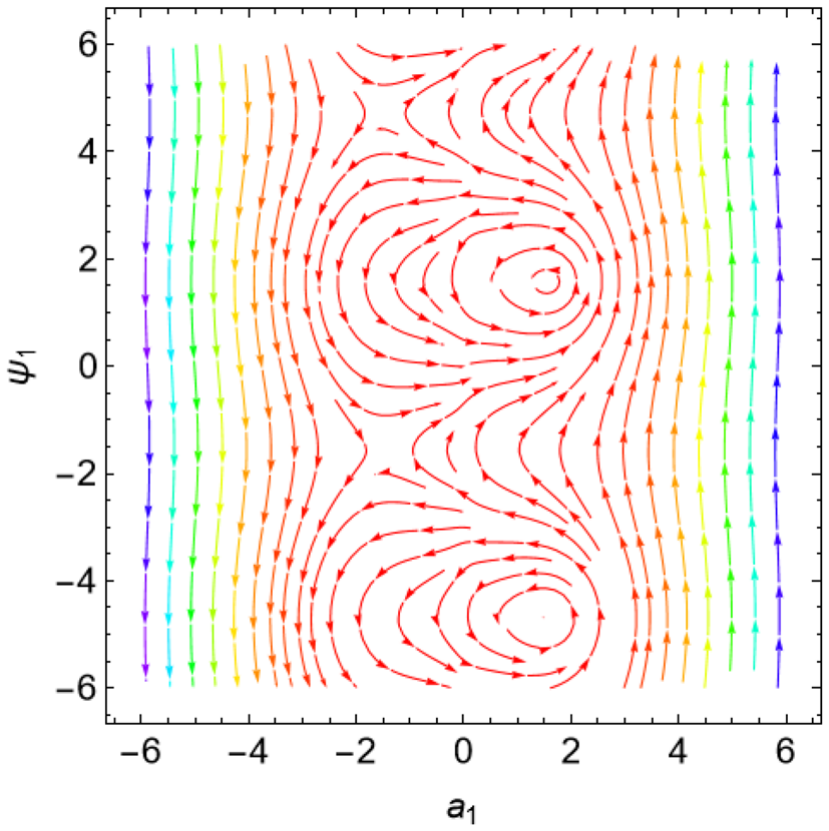
Phase portrait at $\omega = 0.2$.

**Figure 13:**
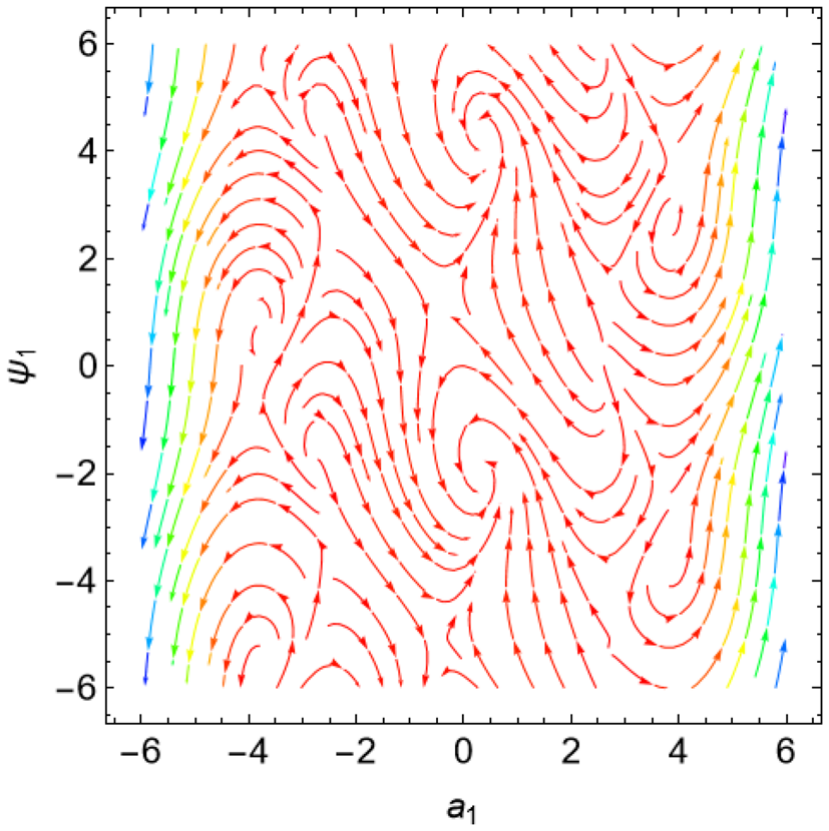
Phase portrait at $\delta = 0.3$.

**Figure 14:**
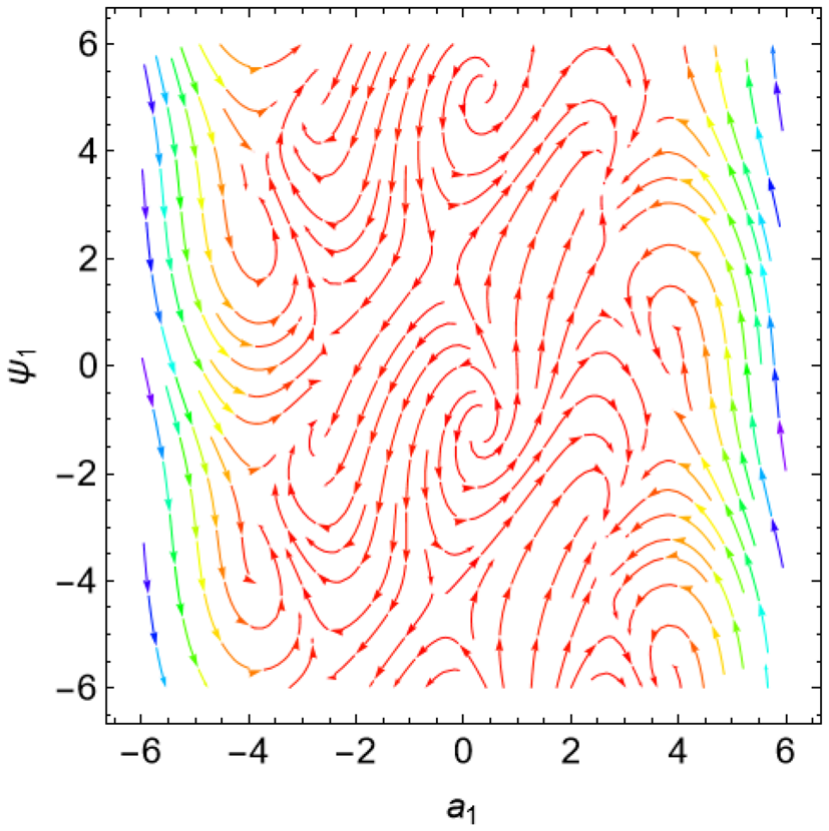
Phase portrait at $\delta = - \,0.3$.

**Figure 15:**
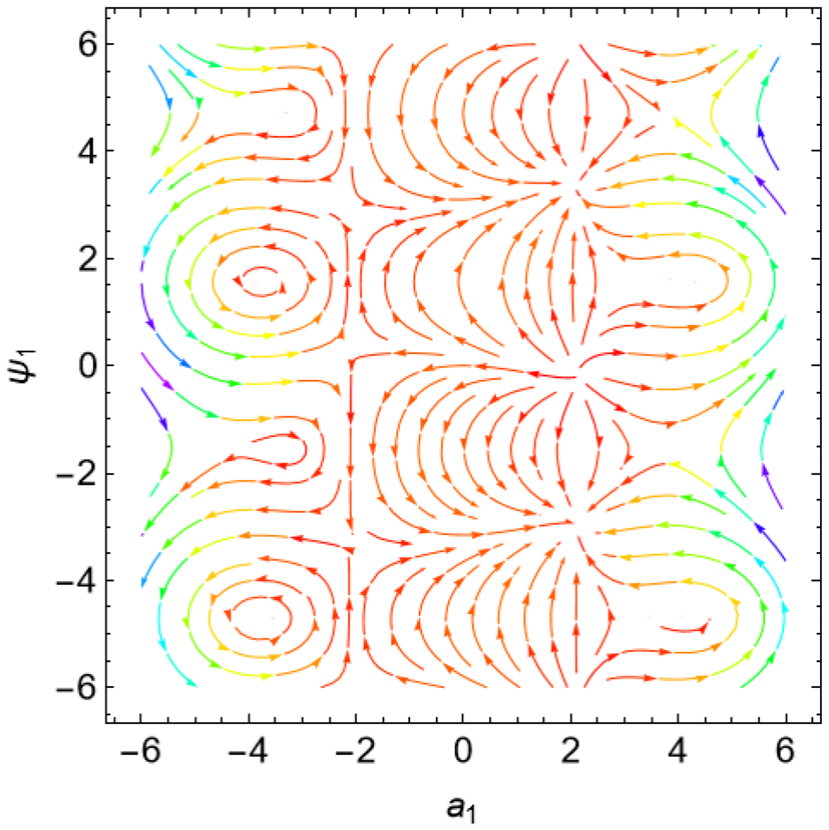
Phase portrait at $F_{1} = 5$.

**Figure 16:**
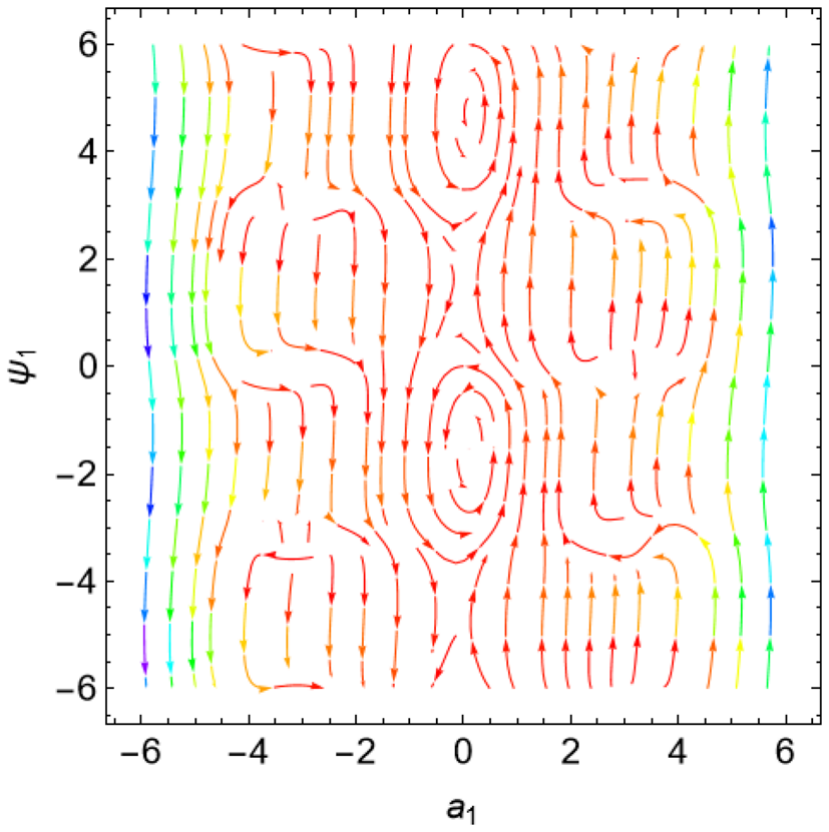
Phase portrait at $F_{3} = 2$.

For more convenience, the corresponding phase portraits are shown below.

### Linearized stability of the non-autonomous system

The steady-state solution of the system through the PPF controller associated to the fixed point of as given in Eqs. (69)–(72) is achieved by considering $\dot{a}_{m} = 0$, and $\dot{\psi }_{m} = 0$,$(m = 1,\,2)$. Therefore, the FREs of the existing case ($a_{1} \ne 0,a_{2} \ne 0$) are given by solving the following algebraic equations:

78 $$\begin{aligned} 0 & = - \frac{\delta }{2}a_{1} + \frac{3\beta \,\delta }{{16\,\omega^{2} }}a_{1}^{3} + \frac{{F_{1} \,(9\beta \,a_{1}^{2} + 8\,\omega \,(\sigma_{1} - 2\omega ))}}{{32\,\omega^{3} }}\cos \psi_{1} + \frac{{\,\delta \,F_{1} }}{{8\,\omega^{2} }}\,\sin \psi_{1} + \frac{{q_{1} \,(\delta - \,\delta_{1} )}}{{8\,\omega^{2} }}a_{2} \,\cos \psi_{2} \\ & \quad \, + \frac{{q_{1} \,( - 9\beta \,a_{1}^{2} + 8\,\omega \,( - \sigma_{2} + 2\omega ))}}{{32\,\omega^{3} }}\,a_{2} \,\sin \psi_{2} - \frac{{F_{2} \,F_{3} }}{4\,\omega \,\Omega \,(2\,\omega - \Omega )}\,a_{1} \,\cos 2\,\psi_{1} + \frac{{(F_{2}^{2} - F_{3}^{2} )}}{8\,\omega \,\Omega \,(2\,\omega - \Omega )}\,a_{1} \,\sin 2\,\psi_{1} , \\ \end{aligned}$$

79 $$\begin{aligned} \sigma_{1} \,a_{1} & = - \frac{{\delta^{2} }}{8\,\omega }\,a_{1} - \frac{{q_{1} \,q_{2} }}{{8\,\omega_{1} \,\omega^{2} }}\,a_{1} + \frac{3\,\beta }{{8\,\omega }}\,a_{1}^{3} - \frac{{15\,\beta^{2} }}{{256\,\omega^{3} }}\,a_{1}^{5} - \frac{{(\,F_{2}^{2} + F_{3}^{2} )}}{4\,\omega \,(2\,\omega - \Omega )\,(2\,\omega + \Omega )}\,a_{1} - \frac{{\delta \,F_{1} }}{{8\,\omega^{2} }}\,\cos \,\psi_{1} \\ & \,\,\,\,\,\,\,\,\,\, + \frac{{F_{1} \,(3\beta \,a_{1}^{2} + 8\,\omega \,(\sigma_{1} - 2\omega ))}}{{32\,\omega^{3} }}\sin \psi_{1} - \frac{{(F_{2}^{2} \, - F_{3}^{2} )}}{8\,\omega \,\Omega \,(2\,\omega - \Omega )}\,a_{1} \,\cos 2\,\psi_{1} - \frac{{F_{2} \,F_{3} }}{4\,\omega \,\Omega \,(2\,\omega - \Omega )}\,a_{1} \,\sin 2\psi_{1} \\ & \,\,\,\,\,\,\,\,\,\, + \frac{{q_{1} \,(\delta - \,\delta_{1} )}}{{8\,\omega^{2} }}a_{2} \,\sin \psi_{2} + \frac{{q_{1} \,(3\beta \,a_{1}^{2} + 8\,\omega \,(\sigma_{2} - 2\omega ))}}{{32\,\omega^{3} }}\,a_{2} \,\cos \psi_{2} , \\ \end{aligned}$$

80 $$0 = - \frac{1}{2}\,\delta_{1} \,a_{2} - \frac{{q_{2} (\delta - \,\delta_{1} )}}{{8\,\omega_{1}^{2} }}\,a_{1} \,\cos \psi_{2} + \frac{{q_{2} (3\beta \,a_{1}^{2} - 8\omega \,\sigma_{2} - 16\,\omega \,\omega_{1} )}}{{32\,\omega \,\omega_{1}^{2} }}\,a_{1} \,\sin \psi_{2} - \frac{{F_{1} \,q_{2} }}{{8\,\omega \,\omega_{1}^{2} }}\,\cos (\psi_{1} - \psi_{2} ),$$

81 $$\begin{aligned} (\sigma_{1} - \sigma_{2} )\,a_{2} & = - \frac{{q_{1} \,q_{2} }}{{8\,\omega \,\omega_{1}^{2} }}\,a_{2} - \frac{{\delta_{1}^{2} }}{{8\,\omega_{1} }}\,a_{2} + \frac{{q_{2} (\delta - \,\delta_{1} )}}{{8\,\omega_{1}^{2} }}\,a_{1} \,\sin \psi_{2} + \frac{{q_{2} (3\,\beta \,a_{1}^{2} - 8\,\omega \,\sigma_{2} - 16\,\omega \,\omega_{1} )}}{{32\,\omega \,\omega_{1}^{2} }}\,a_{1} \,\cos \psi_{2} \\ & \,\,\,\,\,\,\, - \frac{{F_{1} \,q_{2} }}{{8\,\omega \,\omega_{1}^{2} }}\,\sin (\psi_{1} - \psi_{2} ). \\ \end{aligned}$$

To construct the stability configuration of the steady-state solution, consider the following expectations:

82 $$a_{m} = a_{m\,0} + a_{m\,1} ,\quad \psi_{m} = \psi_{m\,0} + \psi_{m\,1} ,\quad (m = 1,\,2),$$

where $a_{m\,0}$ and $\psi_{m\,0}$ are the solutions of Eqs. (69), (71), (73) and (74), and the very small, perturbed quantities are defined by $\psi_{m1}$, and $a_{m1}$. Substituting from Eq. (82) into Eqs. (69), (71), (73) and (74)**,** then keeping only the linear terms of $a_{m\,1}$ and $\psi_{m\,1}$, one gets the following matrix equation:

83 $$\left[ {\begin{array}{*{20}c} {\dot{a}_{11} } \\ {\dot{\psi }_{11} } \\ {\dot{a}_{21} } \\ {\dot{\psi }_{21} } \\ \end{array} } \right] = \left[ {\begin{array}{*{20}c} {r_{11} } & {r_{12} } & {r_{13} } & {r_{14} } \\ {r_{21} } & {r_{22} } & {r_{23} } & {r_{24} } \\ {r_{31} } & {r_{32} } & {r_{33} } & {r_{34} } \\ {r_{41} } & {r_{42} } & {r_{43} } & {r_{44} } \\ \end{array} } \right]\,\left[ {\begin{array}{*{20}c} {a_{11} } \\ {\psi_{11} } \\ {a_{21} } \\ {\psi_{21} } \\ \end{array} } \right],$$

where the above square matrix is called the Jacobian matrix ${\rm X}$. The coefficients values $r_{i\,j}$, ($i,j = 1,2,3,4$) are listed in the Appendix. Therefore, the eigenvalues $\gamma_{r}$, ($r = 1,2,3,4$) are given by ${\rm X} - \gamma_{r} \,I_{4 \times 4} = 0$ and lead to the following equation:

84 $$\gamma^{4} + R_{1} \,\gamma^{3} + R_{2} \,\gamma^{2} + R_{3} \,\gamma + R_{4} = 0,$$

where the coefficients $R_{1}$,$R_{2}$, $R_{3}$ and $R_{4}$ are known from the context. In light of the Routh–Hurwitz criterion^28^, if the real part of the eigenvalue is negative, then the periodic solution is stable; otherwise, it is unstable.

## Results and discussions

### Frequency response curve (FRC) and effect of different factors

In the following Figs. 17, 18, 19, 20, 21, 22, 23, 24, 25, 26 and 27, it should be noted that the thick curves refer to the stable regions. Otherwise, the thin ones represent the unstable regions.

**Figure 17:**
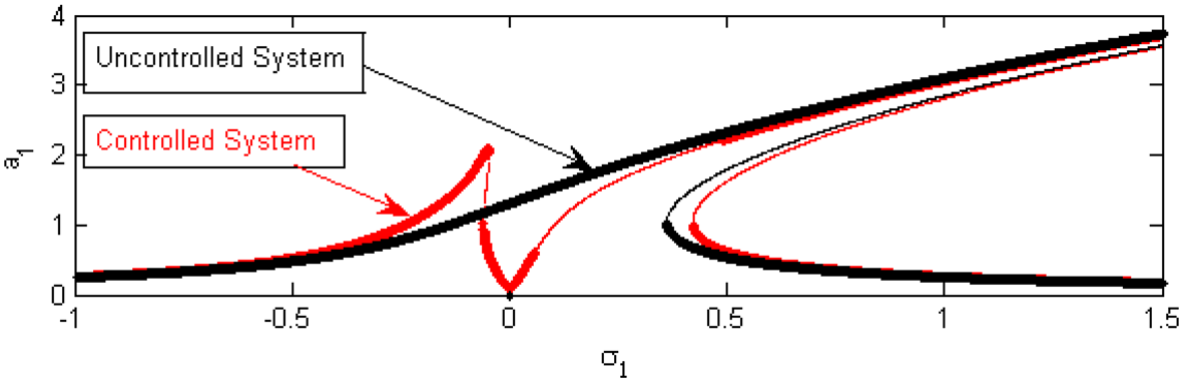
FRC comparison between a controlled system and an uncontrolled system.

**Figure 18:**
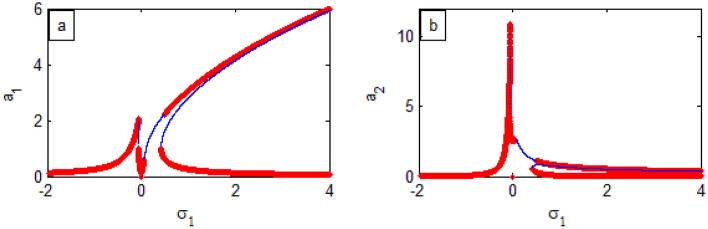
FRC of PPF controlled system (  stable r region), ( unstable region) at *σ*_2_ (**a**) (*a*_1_ against *σ*_1_) and (**b**) (*a*_2_ against *σ*_2_)

**Figure 19:**
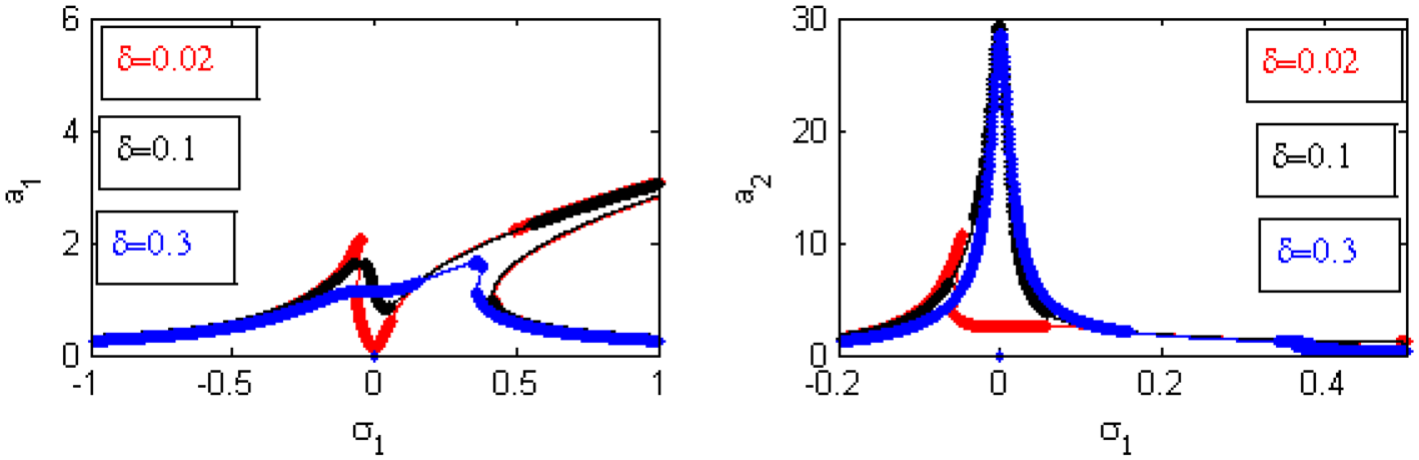
Effects of $\delta$ on FRC.

**Figure 20:**
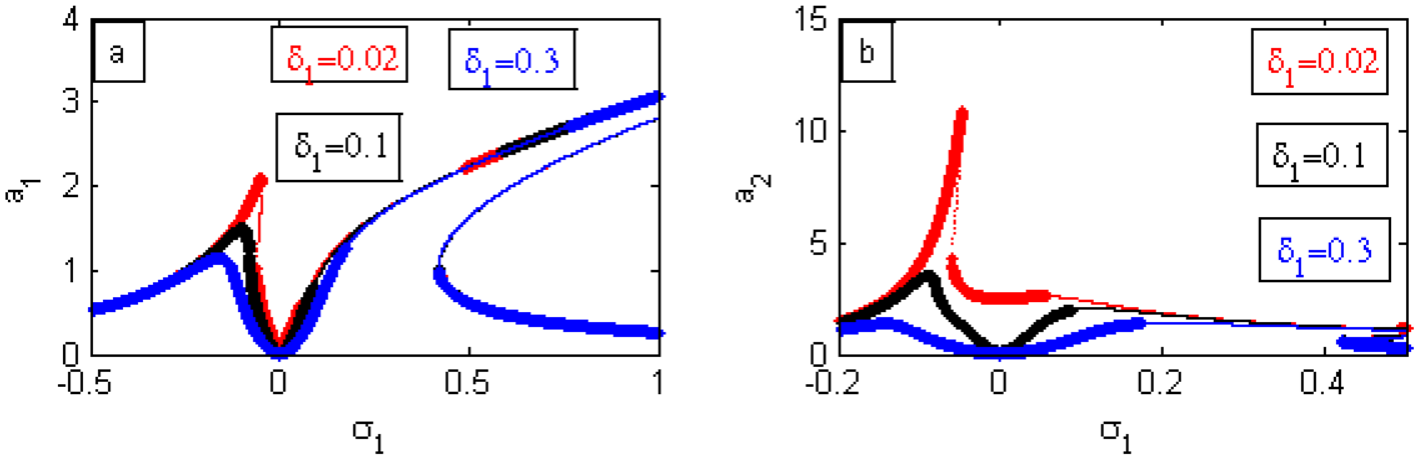
Effects of $\delta_{1}$ on FRC.

**Figure 21:**
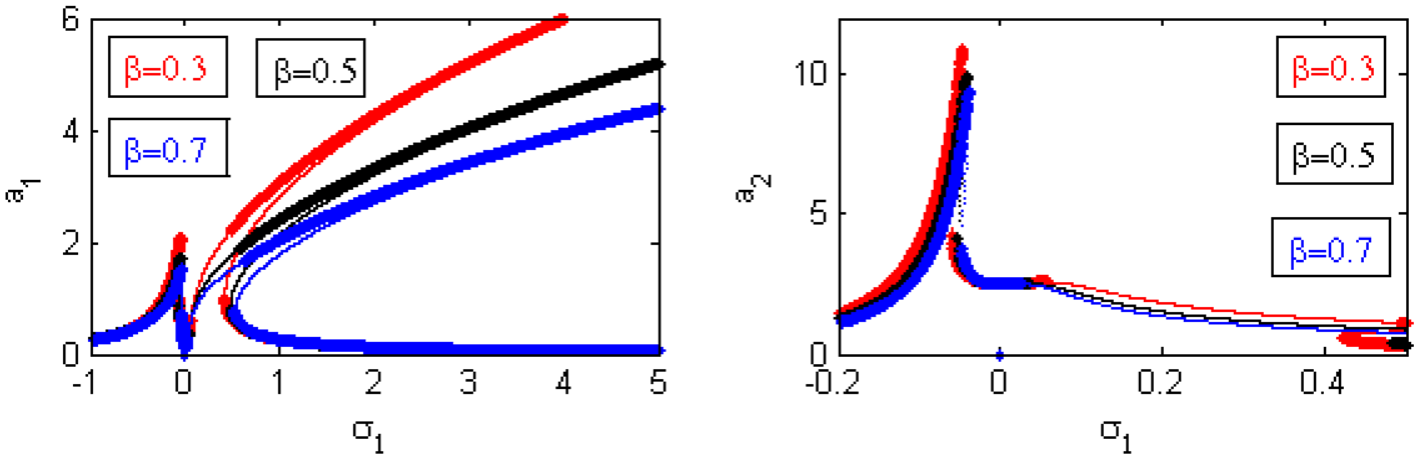
Effects of $\beta$ on FRC.

**Figure 22:**
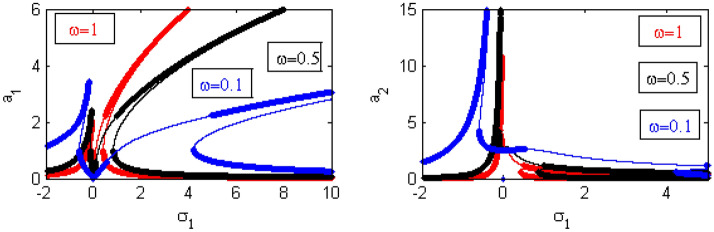
Effects of $\omega$ on FRC.

**Figure 23:**
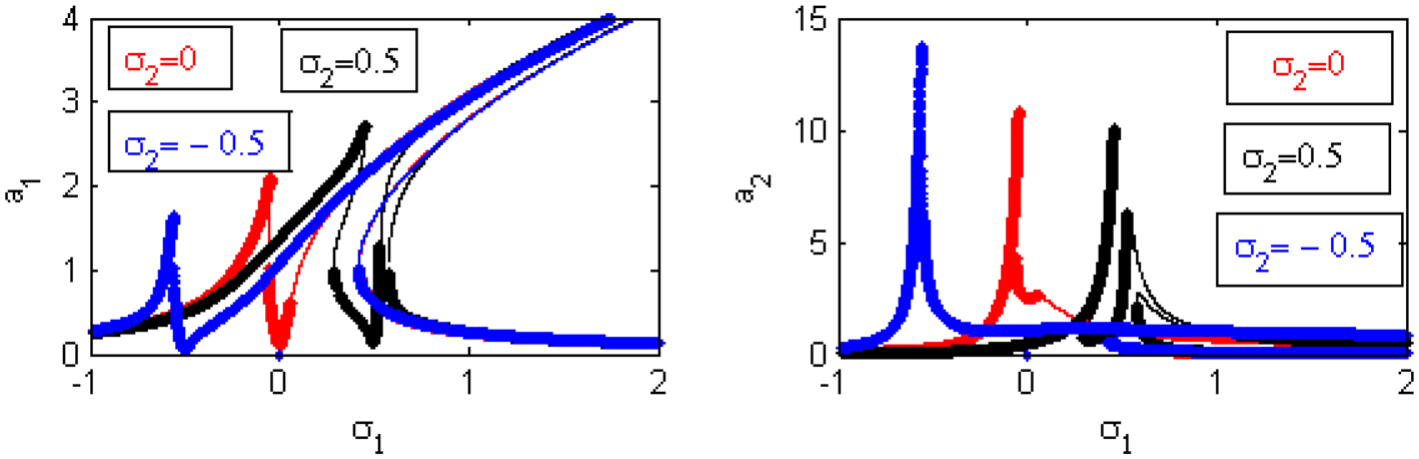
Effects of $\sigma_2$ on FRC.

**Figure 24:**
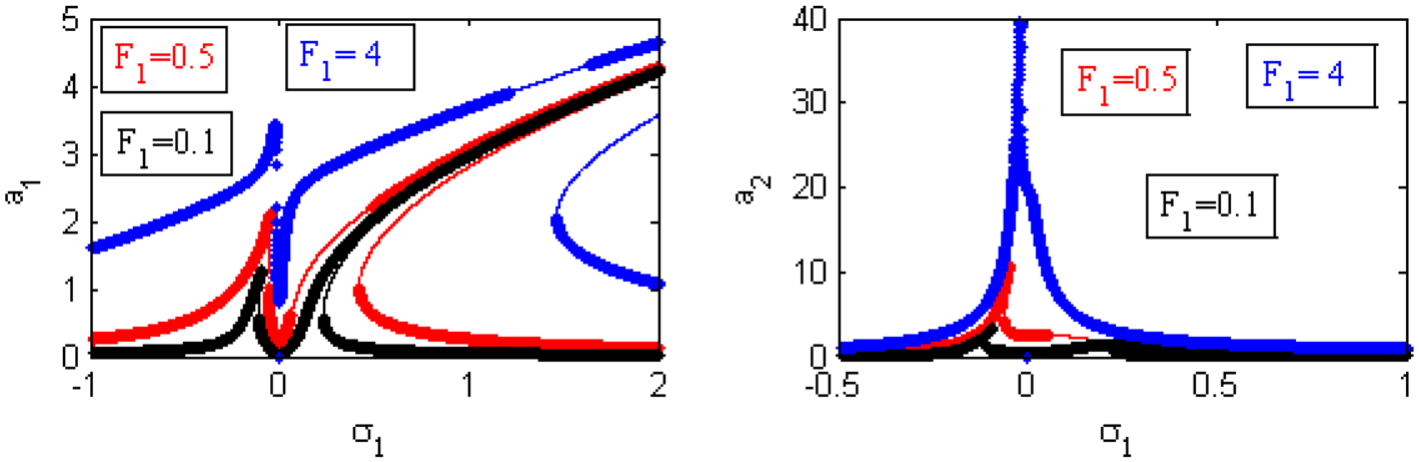
Effects of $F_1$ on FRC.

**Figure 25:**
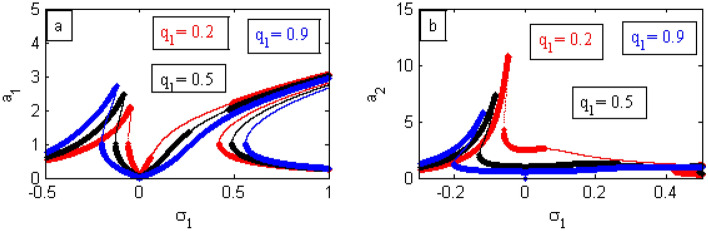
Effects of $q_1$ on FRC.

**Figure 26:**
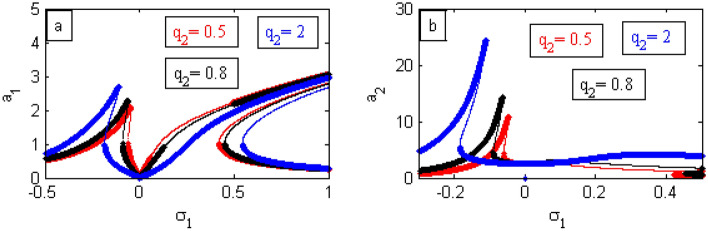
Effects of $q_2$ on FRC.

**Figure 27:**
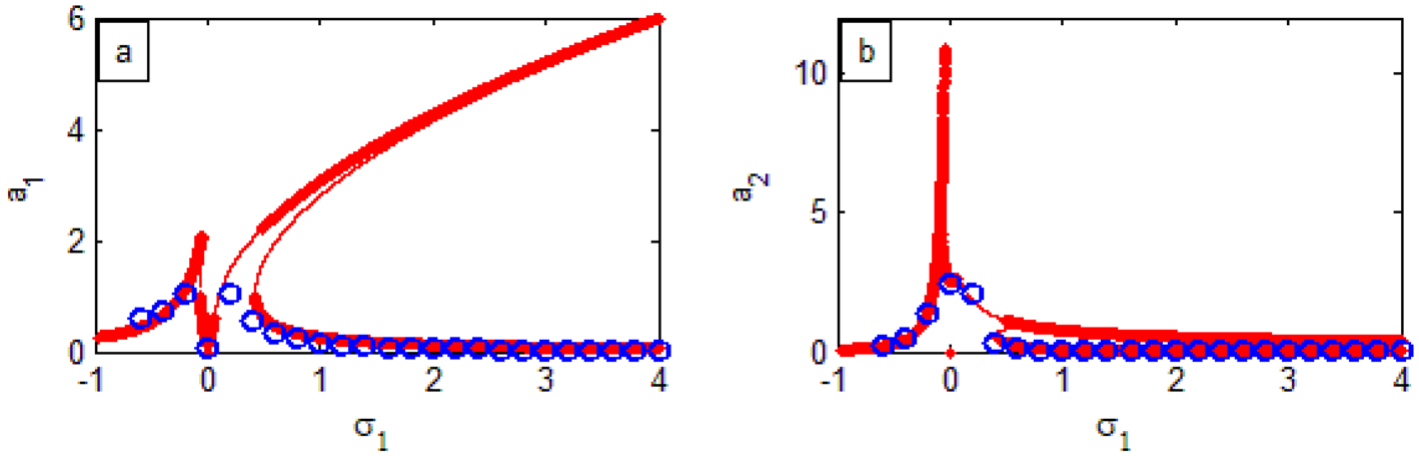
FRC comparison between both RK-4 ( ) and analytic solution ( ).

To clarify the importance of adding the control, we make a comparison between the FRC for the system before and after adding the controller as represented in the Fig. 17. From this comparison, at the region near to $\sigma_{1} = 0$, the steady-state amplitude for the uncontrolled structure is very high as indicated by the black curve. In the same region, the amplitude of the system after adding PPF control decreases completely as indicated by the red curve. This demonstrates the quality of the controller in reducing the vibration at the considered resonance situation. Consequently, when adding the controller, the area around $\sigma_{1} = 0$ is called the vibration bandwidth region because it is located between two peaks as shown earlier^17,34^.

The steady-state solution of the model FREs**,** as given by Eqs. (78)–(81), is described by plotting the FRC at the current case ($a_{1} \ne 0$,$a_{2} \ne 0$) as shown in Fig. 18. For this purpose, Fig. 18a displays the amplitude model $a_{1}$ versus $\sigma_{1}$. Simultaneously, Fig. 18b depicts the controller amplitude $a_{2}$ versus $\sigma_{1}$. Moreover, from Fig. 18a it appears that the vibrations after being connected to the PPF controller are damped in the region of the frequency bandwidth. Consequently, we can say that the optimal mode of vibration reduction is $\sigma_{1} = \sigma_{2} = 0$. Earlier examples were included^21,34^.

Figure 19 illustrates the effects of various amounts of $\delta$ on the FRC. When the values of $\delta$ have increased, the amplitude of the structure has increased around the region $\sigma_{1} = 0$. This is called the jump phenomenon, which was displayed earlier^35,36^. Additionally, the areas of instability and the heights of the two peaks are decreased.

By improving the amounts of $\delta_{1}$, the bandwidth region in Fig. 20a,b are expanded. Moreover, the height of the left peak as well as the instability regions decrease. Finally, the jump phenomenon of the controller decreases as indicated in Fig. 20b. Examples from the previous phenomenon were included in^17^.

From Fig. 21, it is clear that as the values of $\beta$ increase, the amplitude decreases, and the right peak is more bent to the right. Instances from the earlier occurrence were provided earlier^29^.

Figure 22 shows that when the $\omega$-values decrease, the bandwidth region expands, and the regions of instability increase. Additionally, the height of the left peak increases, while the right peak gradually continues to bend to the right. Examples from the first incident were given earlier in^17,21^.

When changing the values of $\sigma_{2}$ as shown in Fig. 23, we note that the lowest values of the steady-state amplitude of the IP occur at $\sigma_{2} = \sigma_{1}$. This establishes that the vibration has been damped at the resonance case. There have already been examples provided from the first instance^17^.

Figure 24 depicts the change of the different values of $F_{1}$ on the FRC curve. It is found that the higher amounts of $F_{1}$ produces the higher amounts of the amplitude and vice-versa for the bandwidth area. Illustrations from the previous situation were already supplied^34^. Therefore, the presence of the magnetic field suppresses the instability of the system.

In what follows, the influences of the parameters $q_{1}$ and $q_{2}$ on the curve FRC will be discussed throughout Figs. 25 and 26. The bandwidth region expands and the left peak goes up when the value of $q_{1}$ and $q_{2}$ are enhanced as displayed in Figs. 25a and 26a. Simultaneously, in Figs. 25b and 26b, the left peak decrease down with an increase of $q_{1}$, while it grows with an increase in $q_{2}$. Similar results were obtained earlier^17,21^.

**Figure 28:**
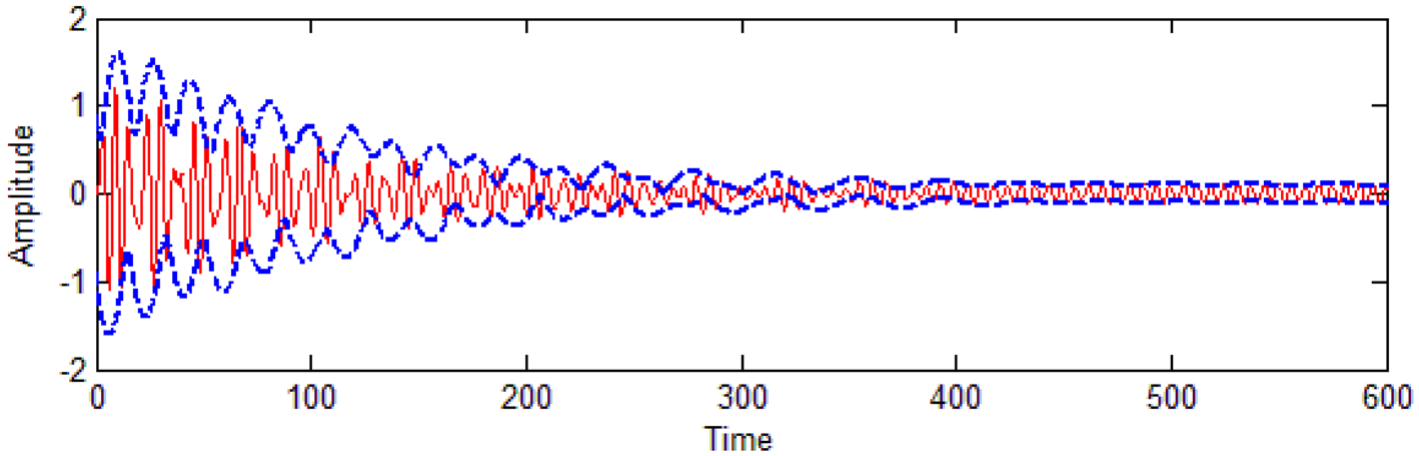
Time history comparison between numerical solution ( ) and an analytic solution ( ) at $\Omega = \omega$ and $\omega_{1} = \omega$.

In the following two figures, a comparison will be made to graphically verify the validation of the theoretical and numerical approaches. For this objective, the comparison of FRC between both analytic and numerical solutions. The solid red lines represent the former, while the blue circles represent the latter as shown in Fig. 27. Fairly good results were obtained (look Tables 3 and 4). It is shown that the analytical solution is very consistent with the numerical one. In Fig. 28, a comparison between the perturbation procedure as given in Eqs. (69), (71), (73) and (74)**,** and the numerical simulation as described in Eqs. (38)–(39) along with the time history was performed. The blue dashed lines show the modulation of the amplitude of the generalized coordinate. Moreover, the red solid lines refer to the time history of vibrations which are simulated numerically of the solutions of the system with PPF-controller. As observed, we found acceptable harmony between numerical and analytical producers, which confirmed our solution as done in Fig. 27.

**Table 3:** Comparison of the obtained results between the RK-4 solution and the analytic solution in Fig. 27a.

$\sigma_{1}$	RK-4 solution	Analytic solution	Absolute error
-0.6	0.5934	0.4345	0.1484
-0.4	0.7176	0.6491	0.0685
-0.2	1.025	1.134	0.109
0.0	0.08807	0.0996	0.0996
0.6	0.3255	0.4684	0.1429
0.8	0.2245	0.3305	.0.106
1.0	0.1667	0.2582	0.0915
1.2	0.1303	0.2128	0.0825
1.4	0.105	0.1814	0.0764
1.6	0.08677	0.1581	0.07133
1.8	0.07298	0.1401	0.06712
2.0	0.0625	0.1259	0.0634
2.2	0.05411	0.1143	0.06019
2.4	0.04716	0.1009	0.05374
2.6	0.04162	0.09655	0.0493
2.8	0.03708	0.0896	0.05252
3.0	0.03333	0.083559	0.050229
3.2	0.02998	0.07832	0.04834
3.4	0.02723	0.0737	0.04647
3.6	0.02477	0.06959	0.04482
3.8	0.0226	0.06559	0.04299
4.0	0.02083	0.0626	0.04177

**Table 4:** Comparison of the obtained results between the RK-4 solution and the analytic solution in Fig. 27b.

$\sigma_{1}$	RK-4 solution	Analytic solution	Absolute Error
-0.6	0.1915	0.1798	0.0117
-0.4	0.5299	0.4056	0.1243
-0.2	1.374	1.415	0.041
0.8	0.05008	0.1003	0.05022
1.0	0.02779	0.06453	0.03674
1.2	0.0169	0.04432	0.02742
1.4	0.01106	0.03238	0.02132
1.6	0.007542	0.0247	0.017158
1.8	0.00534	0.01946	0.014126
2.0	0.003882	0.01574	0.011858
2.2	0.002914	0.01298	0.010066
2.4	0.002239	0.0109	0.008661
2.6	0.001737	0.009284	0.007547
2.8	0.001369	0.008	0.006631
3.0	0.001106	0.006966	0.00586
3.2	0.0009026	0.006118	0.0052154
3.4	0.0007421	0.005419	0.0046769
3.6	0.0006131	0.004833	0.0042199
3.8	0.0005142	0.004335	0.0038208
4.0	0.0004352	0.003913	0.0034778

**Figure 29:**
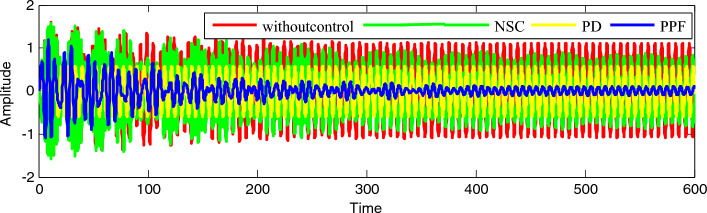
Time history comparison between different controllers.

Figure 29 presents a comparison between different controllers to verify the good performance of the vibration reduction which appears in the IP without a controller described with the red line. The comparison is made with a green line for the nonlinear saturation control (NSC), and with a yellow line for the proportional derivative control (PD), and with a blue line for the PPF.

## Conclusions

In the current work, the vibrating IP system is analyzed analytically and numerically without and with a PPF controller. The approximate solution before the PPF controller is accomplished by applying a modified HPM. A numerical method based on the RK-4 is employed to validate the prior approximate solution. Furthermore, both the phase portraits and the linearized stability are plotted. On the other side, the vibration reduction for IP model via PPF controller is proposed in one of the worst resonance cases. The MSPT technique is used for getting a second-order approximate solution of the controlled measured system. The stability analysis of the situations $\Omega \cong \omega$ and $\omega_{1} \cong \omega$, with the aid of the Routh–Hurwitz criterion, is examined. Moreover, a collection of drawings is made to demonstrate the FRC and different factors using the MATLAB program. The main outcomes of the whole work may be summed in the subsequent points:

1. For both autonomous and non-autonomous examples, phase portraiture and the linearized stability of IP near fixed locations are displayed.
2. The stability of IP is improved by the presence of the magnetic field.
3. The PPF controller is succeeded in reducing vibrations for the considered IP system with a reduced rate of 90.5%.
4. The amplitude of the IP system is increased as the following parameters: $F_{1} ,\omega \,\,{\text{and}}\,\,\delta$ increase, and the bandwidth region progressively decreases for $F_{1} ,\,\omega \,$ and progressively disappears for $\delta$. Additionally, the unstable regions are decreased.
5. The increasing the values of the following parameters $q_{1} ,q_{2} ,\beta \,\,{\text{and}}\,\,\delta_{1}$ leads to a decrease in the amplitude of the IP system progressively.
6. The IP system with a PPF controller is reached to the smallest values on the frequency response curve at $\sigma_{1} = \,\sigma_{2}$.
7. A comparison between the analytical and the numerical scheme provides an appropriate agreement between them before and after adding the PPF controller as presented in Figs. 2 and 28.
8. For validation response curves, there exist great agreements between the approximation FRC and RK-4 solutions as presented in Fig. 27.

For progress works, the topic of IP can be analyzed in different situations: Concerning the approximate analytical solution, and away from the weakness of expanding the restoring forces, a relatively new methodology which is known as the He’s frequency^37–39^ can be applied. Another effective controller could be adopted like a proportional integral derivative (PID) controller or nonlinear integral positive position feedback (NIPPF) controller to decrease the high vibration amplitude of the systems in a small time.

## Data Availability

All data generated or analyzed during this study are included in this manuscript.

## Appendix

The following is a possible list of the coefficients found in Eq. (25):

$$\begin{aligned} & \theta_{11} = \frac{{2\,\delta \,( - 2 + 16\,\sigma^{2} - \Omega^{2} )}}{{L\,(64\,\sigma^{4} - 20\,\sigma^{2} \,\Omega^{2} + \Omega^{4} )}},\quad \theta_{12} = \frac{{\delta \,(1 - 8\,\sigma^{2} )}}{{16\,L\,\sigma^{3} \,(2\,\sigma - \,\Omega )}},\quad \theta_{13} = \frac{{\delta \,(1 - 8\,\sigma^{2} )}}{{16\,L\,\sigma^{3} \,(2\,\sigma + \,\Omega )}}, \\ & \theta_{14} = \frac{\delta \,\Omega }{{48\,L\,\sigma^{3} \,(8\,\sigma^{2} - 6\,\sigma \,\Omega + \Omega^{2} )}},\quad \theta_{15} = \frac{ - \delta \,\Omega }{{48\,L\,\sigma^{3} \,(2\,\sigma + \,\Omega )\,(4\,\sigma + \,\Omega )}},\quad \theta_{16} = \frac{{H\,\Omega \,( - 1 + 4\,\sigma^{2} )}}{{4\,L\,\sigma^{2} \,(\sigma^{2} - \Omega^{2} )}}, \\ & \theta_{17} = \sigma_{171} /\sigma_{172} ,\quad \theta_{18} = \frac{ - 1}{{192\,L\,\sigma^{5} }}, \\ \end{aligned}$$

$$\begin{aligned} \theta_{171} & = - 9216\,\left( {1 + 4\,H} \right)\,\sigma^{10} \,\Omega^{2} - 30\,\sigma^{2} \,\Omega^{6} + \Omega^{8} - 4\,\sigma^{6} \,\Omega^{2} \,\left( \begin{gathered} 205 + 160\,\left( {1 + 2\,H} \right)\,\,\Omega^{2} \hfill \\ + 16\,\left( {26 + 29\,H} \right)\,\Omega^{4} \hfill \\ \end{gathered} \right) \\ & \,\,\,\,\,\,\,\,\, + 64\,\sigma^{8} \,\left( \begin{gathered} 9 + \left( {9 + 64\,H} \right)\,\,\Omega^{2} \hfill \\ + \,\left( {169 + 244\,H} \right)\,\Omega^{4} \hfill \\ \end{gathered} \right) + \sigma^{4} \,\Omega^{4} \,\left( {273 + 64\,\left( {1 + H} \right)\,\Omega^{2} \,\left( {1 + \Omega^{2} } \right)} \right) \\ \end{aligned}$$

$$\theta_{172} = 64\,L\,\sigma^{5} (576\,\sigma^{8} + \,\Omega^{8} - 820\,\sigma^{6} \,\Omega^{2} + 273\,\sigma^{4} \,\Omega^{4} - 30\,\sigma^{2} \,\Omega^{6} )$$

$$\begin{aligned} & \theta_{19} = \frac{{\Omega \,(1 - 8\,\sigma^{2} )}}{{16\,L\,\sigma^{3} \,(2\,\sigma - \,\Omega )}},\quad \theta_{20} = \frac{{\Omega \,( - 1 + 8\,\sigma^{2} )}}{{16\,L\,\sigma^{3} \,(2\,\sigma + \,\Omega )}},\quad \theta_{21} = \frac{H\,\Omega }{{8\,L\,\sigma^{2} \,(3\,\sigma^{2} - 4\,\sigma \,\Omega + \Omega^{2} )}}, \\ & \theta_{22} = \frac{ - \,H\,\Omega }{{8\,L\,\sigma^{2} \,(\sigma + \Omega )\,(3\,\sigma + \Omega )}},\quad \theta_{23} = \frac{{\Omega^{2} }}{{48\,L\,\sigma^{3} \,(8\,\sigma^{2} - 6\,\sigma \,\Omega + \Omega^{2} )}},\quad {\text{and}}\,\,\,\theta_{24} = \frac{{\Omega^{2} }}{{48\,L\,\sigma^{3} \,(2\,\sigma + \Omega )\,(4\,\sigma + \Omega )}}. \\ \end{aligned}$$

Here is a list of the coefficients found in Eqs. (59) and (60):

$$\begin{aligned} & \theta_{25} = \frac{{ - 3\,\,\tilde{\beta }^{2} \,A^{3} \,\overline{A}^{2} }}{{8\,\omega^{2} }} + \frac{{A\,(\tilde{F}_{2}^{2} \, + \tilde{F}_{3}^{2} )}}{{4\,(\omega^{2} - (\Omega + \omega )^{2} )}} + \frac{{A\,(\tilde{F}_{2}^{2} \, + \tilde{F}_{3}^{2} )}}{{4\,(\omega^{2} - (\Omega - \omega )^{2} )}} - 2i\omega \,D_{2} A - \tilde{\delta }\,D_{1} A - D_{1}^{2} A, \\ & \theta_{26} = \frac{{ - 3\,i\,\tilde{\beta }\,\tilde{\delta }\,A^{3} }}{8\,\omega } - \frac{{3\,\,\tilde{\beta }^{2} \,A^{4} \,\overline{A}}}{{4\,\omega^{2} }} - \frac{{9\,i\,\tilde{\beta }\,A^{2} \,D_{1} A}}{4\,\omega },\quad \theta_{27} = \frac{{ - 3\,\,\tilde{\beta }^{2} \,A^{5} }}{{8\,\omega^{2} }}, \\ \end{aligned}$$

$$\begin{aligned} & \theta_{28} = \frac{1}{{2\,\left( {\omega^{2} - (\Omega + \omega )^{2} } \right)}}\left[ \begin{gathered} \tilde{\delta }\,(\Omega + \omega )\,A\,(i\,\tilde{F}_{2} \, + \tilde{F}_{3} ) + 2\,(\tilde{F}_{2} \, - i\,\tilde{F}_{3} ) \hfill \\ (3\tilde{\beta }\,A^{2} \overline{A} + i\,\,(\Omega + \omega )\,D_{1} A) \hfill \\ \end{gathered} \right] + \left[ {\frac{{3\tilde{\beta }\,A^{2} \overline{A}\,(\tilde{F}_{2} - i\,\tilde{F}_{3} )}}{{2\,(\omega^{2} - (\Omega - \omega )^{2} )}}} \right], \\ & \theta_{29} = \frac{1}{{2\,(\omega^{2} - (\Omega - \omega )^{2} )}}\left[ \begin{gathered} (\tilde{F}_{2} \, - i\,\tilde{F}_{3} )(i\,\tilde{\delta }\,(\Omega - \omega )\,\overline{A}\, \hfill \\ + 6\tilde{\beta }\,\overline{A}^{2} A + 2i\,(\Omega - \omega )\,D_{1} \overline{A}) \hfill \\ \end{gathered} \right] + \left[ {\frac{{3\tilde{\beta }\,\overline{A}^{2} A\,(\tilde{F}_{2} - i\,\tilde{F}_{3} )}}{{2\,(\omega^{2} - (\Omega + \omega )^{2} )}}} \right], \\ & \theta_{30} = \frac{{3\tilde{\beta }\,A^{3} (\tilde{F}_{2} - i\,\tilde{F}_{3} )}}{{2\,(\omega^{2} - (\Omega + \omega )^{2} )}} - \frac{{\tilde{\beta }\,A^{3} (\tilde{F}_{2} - i\,\tilde{F}_{3} )}}{{16\,\omega^{2} }},\quad \theta_{31} = \frac{{3\tilde{\beta }\,\overline{A}^{3} (\tilde{F}_{2} - i\,\tilde{F}_{3} )}}{{2\,(\omega^{2} - (\Omega - \omega )^{2} )}} - \frac{{\tilde{\beta }\,\overline{A}^{3} (\tilde{F}_{2} - i\,\tilde{F}_{3} )}}{{16\,\omega^{2} }}, \\ & \theta_{32} = \frac{{A(\tilde{F}_{2} - i\,\tilde{F}_{3} )^{2} }}{{4\,(\omega^{2} - (\Omega + \omega )^{2} )}},\quad \theta_{33} = \frac{{\overline{A}(\tilde{F}_{2} - i\,\tilde{F}_{3} )^{2} }}{{4\,(\omega^{2} - (\Omega - \omega )^{2} )}}. \\ \end{aligned}$$,

$$\begin{aligned} & \theta_{34} = \frac{{\tilde{q}_{2} \,( - \tilde{F}_{2} + i\,\tilde{F}_{3} )\,\overline{A}}}{{2\,(\omega^{2} - (\Omega - \omega )^{2} )}},\quad \theta_{35} = \frac{{\tilde{q}_{2} \,( - \tilde{F}_{2} + i\,\tilde{F}_{3} )\,A}}{{2\,(\omega^{2} - (\Omega + \omega )^{2} )}},\quad \theta_{36} = \frac{{\tilde{q}_{2} \,\tilde{\beta }\,A^{3} }}{{8\,\omega^{2} }}, \\ & \theta_{37} = - 2\,i\omega_{1} \,D_{2} B\, - \tilde{\delta }_{1} \,D_{1} B - \,D_{1}^{2} B. \\ \end{aligned}$$

$$\begin{aligned} r_{11} & = - \frac{\delta }{2} + \frac{9\,\beta \,\delta }{{16\,\omega^{2} }}a_{10}^{2} + \frac{{9\,F_{1} \,\beta \,}}{{16\,\omega^{3} }}\,a_{10} \cos \psi_{10} + \frac{{(F_{2}^{2} - F_{3}^{2} )}}{8\,\omega \,\Omega \,(2\,\omega - \Omega )}\,\sin \,2\,\psi_{10} \\ &\quad- \frac{{F_{2} \,F_{3} }}{4\,\omega \,\Omega \,(2\,\omega - \Omega )}\,\cos 2\,\psi_{10} - \frac{{9\,q_{1} \,\beta \,}}{{16\,\omega^{3} }}\,a_{10} \,a_{20} \,\sin \psi_{20} , \end{aligned}$$

$$\begin{aligned} r_{12} & = \frac{{\delta \,F_{1} }}{{8\,\omega^{2} }}\,\cos \theta_{10} + \frac{{F_{1} }}{2\,\omega }\,\sin \theta_{10} - \frac{{9\,F_{1} \,\beta \,a_{10}^{2} }}{{32\,\omega^{3} }}\sin \psi_{10} + \frac{{(F_{2}^{2} - F_{3}^{2} )}}{4\,\omega \,\Omega \,(2\,\omega - \Omega )}\,a_{10} \,\cos^{2} \,\psi_{10} - \frac{{(F_{2}^{2} - F_{3}^{2} )}}{4\,\omega \,\Omega \,(2\,\omega - \Omega )}\,a_{10} \,\sin^{2} \,\psi_{10} \\ & \,\,\,\,\,\,\, + \frac{{F_{2} \,F_{3} }}{2\,\omega \,\Omega \,(2\,\omega - \Omega )}\,a_{10} \,\sin 2\,\psi_{10} - \frac{{F_{1} \,\sigma_{1} }}{{4\,\omega^{2} }}\,\sin \theta_{10} , \\ \end{aligned}$$

$$r_{13} = \frac{{\delta \,q_{1} }}{{8\,\omega^{2} }}\,\cos \psi_{20} + \frac{{q_{1} }}{2\,\omega }\,\sin \psi_{20} - \frac{{9\,q_{1} \,\beta \,}}{{32\,\omega^{3} }}\,a_{10}^{2} \,\sin \psi_{20} - \frac{{\delta_{1} \,q_{1} }}{{8\,\omega^{2} }}\,\cos \psi_{20} - \frac{{\sigma_{2} \,q_{1} }}{{4\,\omega^{2} }}\,\sin \psi_{20} ,$$

$$r_{14} = - \frac{{\delta \,q_{1} }}{{8\,\omega^{2} }}\,a_{20} \,\sin \psi_{20} + \frac{{q_{1} }}{2\,\omega }a_{20} \,\cos \psi_{20} - \frac{{9\,q_{1} \,\beta \,}}{{32\,\omega^{3} }}\,a_{10}^{2} \,a_{20} \,\cos \psi_{20} + \frac{{\delta_{1} \,q_{1} }}{{8\,\omega^{2} }}\,a_{20} \,\sin \psi_{20} - \frac{{\sigma_{2} \,q_{1} }}{{4\,\omega^{2} }}\,a_{20} \,\cos \psi_{20} ,$$

$$\begin{aligned} r_{21} & = \frac{{\delta^{2} }}{{8\,\omega \,a_{10} }} - \frac{9\,\beta \,}{{8\,\omega }}a_{10} + \frac{{75\,\beta^{2} }}{{256\,\omega^{3} }}a_{10}^{3} - \frac{{3\,\beta \,F_{1} }}{{16\,\omega^{3} }}\,\sin \psi_{10} + \frac{{(\,F_{2}^{2} + F_{3}^{2} )}}{{4\,\omega \,(2\,\omega - \Omega )\,(2\,\omega + \Omega )\,a_{10} }} + \frac{{(F_{2}^{2} \, - F_{3}^{2} )}}{{8\,\omega \,\Omega \,(2\,\omega - \Omega )\,a_{10} }}\,\cos^{2} \,\psi_{10} \\ & \,\,\,\,\,\,\,\,\,\, - \frac{{(F_{2}^{2} \, - F_{3}^{2} )}}{{8\,\omega \,\Omega \,(2\,\omega - \Omega )\,a_{10} }}\,\sin^{2} \,\psi_{10} + \frac{{F_{2} \,F_{3} }}{{4\,\omega \,\Omega \,(2\,\omega - \Omega )\,a_{10} }}\,\sin 2\psi_{10} - \frac{{3\,q_{1} \,\beta }}{{16\,\omega^{3} }}\,a_{20} \,\cos \psi_{20} + \frac{{\sigma_{1} }}{{a_{10} }} + \frac{{q_{1} \,q_{2} }}{{8\,\omega^{2} \,\omega_{1} \,a_{10} }}, \\ \end{aligned}$$

$$\begin{aligned} r_{22} & = \frac{{F_{1} }}{{2\,\omega \,a_{10} }}\,\cos \psi_{10} - \frac{{\delta \,F_{1} }}{{8\,\omega^{2} \,a_{10} }}\,\sin \psi_{10} - \frac{{3\,\beta \,F_{1} }}{{32\,\omega^{3} }}\,a_{10} \,\cos \psi_{10} - \frac{{(F_{2}^{2} \, - F_{3}^{2} )}}{4\,\omega \,\Omega \,(2\,\omega - \Omega )}\,\sin \,2\,\psi_{10} \\ & \,\,\,\,\,\,\,\,\,\, + \frac{{F_{2} \,F_{3} }}{2\,\omega \,\Omega \,(2\,\omega - \Omega )}\,\cos 2\,\psi_{1} - \frac{{\sigma_{1} \,F_{1} }}{{4\,\omega^{2} \,a_{10} }}\,\cos \psi_{10} , \\ \end{aligned}$$

$$r_{23} = \frac{{q_{1} }}{{2\,\omega \,a_{10} }}\,\cos \psi_{20} - \frac{{\delta \,q_{1} }}{{8\,\omega^{2} \,a_{10} }}\,\sin \psi_{20} - \frac{{3\,\beta \,q_{1} }}{{32\,\omega^{3} }}\,a_{10} \,\cos \psi_{20} + \frac{{q_{1} \,\delta_{1} }}{{8\,\omega^{2} \,a_{10} }}\,\sin \psi_{20} - \frac{{q_{1} \,\sigma_{2} }}{{4\,\omega^{2} \,a_{10} }}\,\cos \psi_{20} ,$$

$$r_{24} = - \frac{{q_{1} \,a_{20} }}{{2\,\omega \,a_{10} }}\,\sin \psi_{20} - \frac{{\delta \,q_{1} \,a_{20} }}{{8\,\omega^{2} \,a_{10} }}\,\cos \psi_{20} + \frac{{3\,\beta \,q_{1} }}{{32\,\omega^{3} }}\,a_{10} \,a_{20} \,\sin \psi_{20} + \frac{{q_{1} \,\delta_{1} \,a_{20} }}{{8\,\omega^{2} \,a_{10} }}\,\cos \psi_{20} + \frac{{q_{1} \,\sigma_{2} \,a_{20} }}{{4\,\omega^{2} \,a_{10} }}\,\sin \psi_{20} ,$$

$$r_{31} = - \frac{{q_{2} (\delta - \,\delta_{1} )}}{{8\,\omega_{1}^{2} }}\,\cos \psi_{20} + \frac{{9\,q_{2} \beta }}{{32\,\omega \,\omega_{1}^{2} }}\,a_{10}^{2} \,\sin \psi_{20} - \frac{{q_{2} \,\sigma_{2} }}{{4\,\omega_{1}^{2} }}\,\sin \psi_{20} - \frac{{q_{2} }}{{2\,\omega_{1} }}\,\sin \psi_{20} ,$$

$$r_{32} = \frac{{F_{1} \,q_{2} }}{{8\,\omega \,\omega_{1}^{2} }}\,\cos \psi_{20} \,\sin \psi_{10} - \frac{{F_{1} \,q_{2} }}{{8\,\omega \,\omega_{1}^{2} }}\,\cos \psi_{10} \,\sin \psi_{20} ,\quad r_{33} = - \frac{1}{2}\,\delta_{1} ,$$

$$\begin{aligned} r_{34} & = \frac{{q_{2} (\delta - \,\delta_{1} )}}{{8\,\omega_{1}^{2} }}\,a_{10} \,\sin \psi_{20} + \frac{{3\,q_{2} \,\beta }}{{32\,\omega \,\omega_{1}^{2} }}\,a_{10}^{3} \,\cos \psi_{20} - \frac{{F_{1} \,q_{2} }}{{8\,\omega \,\omega_{1}^{2} }}\,\cos \psi_{20} \,\sin \psi_{10} + \frac{{F_{1} \,q_{2} }}{{8\,\omega \,\omega_{1}^{2} }}\,\cos \psi_{10} \,\sin \psi_{20} \\ & \,\,\,\,\,\, - \frac{{q_{2} \,\delta_{1} }}{{8\,\omega_{1}^{2} }}\,a_{10} \,\sin \psi_{20} - \frac{{q_{2} \,\sigma_{2} }}{{4\,\omega_{1}^{2} }}\,a_{10} \,\cos \psi_{20} - \frac{{q_{2} }}{{2\,\omega_{1} }}\,a_{10} \,\cos \psi_{20} , \\ \end{aligned}$$

$$\begin{aligned} r_{41} & = \frac{{\delta^{2} }}{{8\,\omega \,a_{10} }} - \frac{9\,\beta }{{8\,\omega }}\,a_{10} + \frac{{75\,\,\beta^{2} }}{{256\,\omega^{3} }}\,a_{10}^{3} - \frac{{3\,\beta \,F_{1} }}{{16\,\omega^{3} }}\,\sin \psi_{10} + \frac{{(\,F_{2}^{2} + F_{3}^{2} )}}{{4\,\omega \,(2\,\omega - \Omega )\,(2\,\omega + \Omega )\,a_{10} }} - \frac{{3\,\beta \,q_{1} }}{{16\,\omega^{3} }}\,a_{20} \,\cos \psi_{20} + \frac{{\sigma_{1} }}{{a_{10} }} \\ & \,\,\,\,\,\,\, + \frac{{(F_{2}^{2} \, - F_{3}^{2} )}}{{8\,\omega \,\Omega \,(2\,\omega - \Omega )\,a_{10} }}\,\cos 2\,\psi_{10} + \frac{{F_{2} \,F_{3} }}{{4\,\omega \,\Omega \,(2\,\omega - \Omega )\,a_{10} }}\,\sin 2\psi_{10} + \frac{{\delta \,q_{2} }}{{8\,\omega_{1}^{2} \,a_{20} }}\,\sin \psi_{20} + \frac{{9\,\beta \,q_{2} }}{{32\,\omega \,\omega_{1}^{2} \,a_{20} }}\,a_{20}^{2} \,\cos \psi_{20} \\ & \,\,\,\,\,\,\, - \frac{{\delta_{1} \,q_{2} }}{{8\,\omega_{1}^{2} \,a_{20} }}\,\sin \psi_{20} - \frac{{\sigma_{2} \,q_{2} }}{{4\,\omega_{1}^{2} \,a_{20} }}\,\cos \psi_{20} - \frac{{q_{2} }}{{2\,\omega_{1} \,a_{20} }}\,\cos \psi_{20} + \frac{{q_{1} \,q_{2} }}{{8\,\omega_{1}^{2} \,a_{10} }}, \\ \end{aligned}$$

$$\begin{aligned} r_{42} & = \frac{{F_{1} }}{{2\,\omega \,a_{10} }}\,\cos \psi_{10} - \frac{{\delta \,F_{1} }}{{8\,\omega^{2} \,a_{10} }}\,\sin \psi_{10} - \frac{{3\,\beta \,F_{1} }}{{32\,\omega^{3} }}\,a_{10} \,\cos \psi_{10} - \frac{{(F_{2}^{2} \, - F_{3}^{2} )}}{4\,\omega \,\Omega \,(2\,\omega - \Omega )\,}\,\sin 2\,\psi_{10} \\ & 
\,\,\,\,\,\,\, + \frac{{F_{2} \,F_{3} }}{2\,\omega \,\Omega \,(2\,\omega - \Omega )}\,\cos 2\psi_{10} - \frac{{F_{1} \,\sigma_{1} }}{{4\,\omega^{2} \,a_{10} }}\,\cos \psi_{10} - \frac{{F_{1} \,q_{2} }}{{8\,\omega \,\omega_{1}^{2} \,a_{20} }}\,\cos (\psi_{10} - \psi_{20} ), \\ \end{aligned}$$

$$\begin{aligned} r_{43} & = \frac{{\delta^{2} }}{{8\,\omega \,a_{20} }} - \frac{3\,\beta }{{8\,\omega \,a_{20} }}\,a_{10}^{2} + \frac{{15\,\,\beta^{2} }}{{256\,\omega^{3} \,a_{20} }}\,a_{10}^{4} + \frac{{\delta \,F_{1} }}{{8\,\omega^{2} \,a_{10} \,a_{20} }}\,\cos \psi_{10} + \frac{{F_{1} }}{{2\,\omega \,a_{10} \,a_{20} }}\,\sin \psi_{10} - \frac{{3\,\beta \,F_{1} }}{{32\,\omega^{3} \,a_{20} }}\,a_{10} \,\,\sin \psi_{10} \\ & \,\,\,\,\,\,\, + \frac{{(\,F_{2}^{2} + F_{3}^{2} )}}{{4\,\omega \,(2\ ,\omega - \Omega )\,(2\,\omega + \Omega )\,a_{20} }} + \frac{{(F_{2}^{2} \, - F_{3}^{2} )}}{{8\,\omega \,\Omega \,(2\,\omega - \Omega )\,a_{20} }}\,\cos 2\,\psi_{10} + \frac{{F_{2} \,F_{3} }}{{4\,\omega \,\Omega \,(2\,\omega - \Omega )\,a_{20} }}\,\sin 2\psi_{10} + \frac{{q_{1} }}{{\omega \,a_{10} }}\,\cos \psi_{20} \\ & \,\,\,\,\,\,\,\, - \frac{{\delta \,q_{1} }}{{4\,\omega^{2} \,a_{10} }}\,\sin \psi_{20} - \frac{{3\,\beta \,q_{1} }}{{16\,\omega^{3} }}\,a_{10} \,\cos \psi_{20} + \frac{{\delta_{1} \,q_{1} }}{{4\,\omega^{2} \,a_{10} }}\,\sin \psi_{20} - \frac{{F_{1} \,\sigma_{1} }}{{4\,\omega^{2} \,a_{10} \,a_{20} }}\,\sin \psi_{10} + \frac{{\sigma_{2} }}{{a_{20} }} - \frac{{\sigma_{2} \,q_{1} }}{{2\,\omega^{2} \,a_{10} }}\,\cos \psi_{20} \\ & \,\,\,\,\,\,\, - \frac{{q_{1} \,q_{2} }}{{8\,\omega \,\omega_{1}^{2} \,a_{20} }} + \frac{{q_{1} \,q_{2} }}{{8\,\omega_{1} \,\omega^{2} \,a_{20} }} - \frac{{\delta_{1}^{2} }}{{8\,\omega_{1} \,a_{20} }},\,\,\,\,{\text{and}} \\ \end{aligned}$$

and $$\begin{gathered} r_{44} = - \frac{{\delta \,q_{1} }}{{8\,\omega^{2} \,a_{10} }}\,a_{20} \,\cos \psi_{20} - \frac{{q_{1} }}{{2\,\omega \,a_{10} }}\,a_{20} \,\sin \psi_{20} + \frac{{3\,\beta \,q_{1} }}{{32\,\omega^{3} }}\,a_{10} \,a_{20} \,\sin \psi_{20} + \frac{{\delta_{1} \,q_{1} }}{{8\,\omega^{2} \,a_{10} }}\,a_{20} \,\cos \psi_{20} + \frac{{\sigma_{2} \,q_{1} }}{{4\,\omega^{2} \,a_{10} }}\,a_{20} \,\sin \psi_{20} \hfill \\ \,\,\,\,\,\,\,\, + \frac{{\delta \,q_{2} }}{{8\,\omega^{2} \,a_{20} }}\,a_{10} \,\cos \psi_{20} - \frac{{3\,\beta \,q_{2} }}{{32\,\omega \,\omega_{1}^{2} \,a_{20} }}\,a_{10}^{3} \,\sin \psi_{20} + \frac{{F_{1} \,q_{2} }}{{8\,\omega \,\omega_{1}^{2} \,a_{20} }}\,\cos (\psi_{10} + \psi_{20} ) - \frac{{\delta_{1} \,q_{2} }}{{8\,\omega_{1}^{2} \,a_{20} }}\,a_{10} \,\cos \psi_{20} \hfill \\ \,\,\,\,\,\,\,\, + \frac{{\sigma_{2} \,q_{2} }}{{4\,\omega_{1}^{2} \,a_{20} }}\,a_{10} \,\sin \psi_{20} + \frac{{q_{2} }}{{2\,\omega_{1} \,a_{20} }}\,a_{10} \,\sin \psi_{20} \hfill \\ \end{gathered}$$
